# *Bacteroides uniformis*-induced perturbations in colonic microbiota and bile acid levels inhibit TH17 differentiation and ameliorate colitis developments

**DOI:** 10.1038/s41522-023-00420-5

**Published:** 2023-08-14

**Authors:** YiTing Yan, Yu Lei, Ying Qu, Zhen Fan, Ting Zhang, Yangbin Xu, Qian Du, Daniel Brugger, Yulin Chen, Ke Zhang, Enping Zhang

**Affiliations:** 1 Key Laboratory of Animal Genetics, Breeding and Reproduction of Shaanxi Province, College of Animal Science and Technology, Northwest A&F University, Yangling, 712100 China; 2 College of Veterinary Medicine, Northwest A&F University, Yangling, 712100 China; 3 Institute of Animal Nutrition and Dietetics, Vetsuisse-Faculty, University of Zurich, 8057 Zurich, Switzerland

**Keywords:** Microbiology, Cellular microbiology

## Abstract

Inflammatory bowel disease (IBD) is associated with gut dysbiosis and can lead to colitis-associated malignancies. *Bacteroides uniformis* (Bu) regulates animal intestinal homeostasis; however, the mechanism by which it alleviates colitis in mice remains unknown. We investigated the effects of *B. uniformis* JCM5828 and its metabolites on female C57BL/6J mice with dextran sulfate sodium salt (DSS) induced colitis. Treatment with Bu considerably alleviated colitis progression and restored the mechanical and immune barrier protein expression. Additionally, Bu increased the abundance of the symbiotic bacteria *Bifidobacterium* and *Lactobacillus vaginalis* while decreasing that of pathogenic *Escherichia-Shigella*, and modulated intestinal bile acid metabolism. Bu largely regulated the expression of key regulatory proteins of the NF-κB and mitogen-activated protein kinase (MAPK) signaling pathways in colonic tissues and the differentiation of TH17 cells. However, Bu could not directly inhibit TH17 cell differentiation in vitro; it modulated the process in the lamina propria by participating in bile acid metabolism and regulating key metabolites (alpha-muricholic, hyodeoxycholic, and isolithocholic acid), thereby modulating the intestinal immune response. Our findings suggest that Bu or bile acid supplements are potential therapies for colitis and other diseases associated with intestinal barrier dysfunction.

## Introduction

Inflammatory bowel disease (IBD) is a chronic, nonspecific inflammatory disease of unknown etiology. IBD mainly includes ulcerative colitis (UC) and Crohn’s disease (CD) and is mostly common in the colon and rectum, with lesions confined to the mucosa and submucosa of the large intestine^1^. IBD has a protracted and recurrent course and affects 10 out of 10,000 people worldwide^2^. Currently available therapies for IBD include non-targeted therapies, such as immunomodulators, aminosalicylic acid, and glucocorticoids, and targeted therapies, such as anti-TNF, anti-IL-12 /IL-23, and anti-α4β7 integrin^3–5^. Adverse events during infancy can cause dysregulation of host intestinal microecology, resulting in the onset of IBD^6^. IBD most frequently manifests as dysregulation of the metabolism of tryptophan, bile acids, and short-chain fatty acids (SCFAs)^7^. Patients with IBD have reduced intestinal SCFA levels and bile salt hydrolase activity. SCFAs can regulate mucosal immunity by promoting B-cell development, Treg differentiation and expansion, inflammatory vesicle activation, and IL-18 production^8^. Furthermore, bile acids can induce an immunomodulatory response by activating receptors such as farnesoid X receptor (FXR). Reduced bile salt hydrolase activity disrupts the primary and secondary bile acid balance in IBD patients^9^. Gut microbes regulate the abundance of key metabolites^10,11^. This suggests using microbes to synthesize these molecules with high efficiency as a means of preventing and treating IBD.

*Bacteroides* are the most prevalent and abundant members of the mammalian gut microbiota. *Bacteroides* provide colonization resistance to gut pathogens^12^. Furthermore, they compete with pathogens for host-derived amino acids (proline and hydroxyproline) and monosaccharides (ribose, fucose, arabinose, rhamnose, and fructose) and produce SCFAs, thereby directly inhibiting pathogenesis^13^. Additionally, the evolutionarily conserved enzyme class B-Hex, expressed by *Bacteroides* in the mouse intestine, drives the differentiation of CD8aa-expressing intraepithelial lymphocytes (CD4IEL). B-Hex-specific lymphocytes suppress inflammation and protect the intestine in a mouse IBD model^14^. In addition, *B. thetaiotaomicron* produces bacterial extracellular vesicles (BEVs) on dendritic cells, macrophages, and monocytes. Once released into the lumen of the intestine, BEVs cross the mucus layer and enter the underlying immune cells, thereby suppressing intestinal inflammation^15^. *B. fragilis* can also prevent colon tumorigenesis, mainly by producing polysaccharide A. This process is mediated by TLR2 signaling, inhibiting the expression of the chemokine receptor CCR5 in the colon^16^. Furthermore, Bu is significantly and positively related to the reduction of early weaning-induced colonic inflammation in mammals^17^. However, whether Bu reduces the incidence of chronic intestinal inflammation remains unknown.

Recent genomic studies have demonstrated that β-glucans can be utilized by Bu in the gut and may be shared with human gut bacteria to maintain gut microbial homeostasis^18^. Furthermore, Bu has been shown to prevent or decrease the incidence of long-lasting IBD in humans, highlighting its potential to enhance microbiota growth and durability. Specifically, Bu has been found to restore the ratio of lymphocytes to type 3 innate lymphoid cells in the intestinal epithelium, which reverses the metabolic and immune alterations that contribute to diet-induced obesity^19,20^. Despite these findings, the probiotic processes of Bu remain unclear, particularly regarding its crucial metabolites and targeted cells. Therefore, it is necessary to determine the mechanisms of action of Bu in treating IBD to promote the use of this probiotic for the benefit of IBD patients. This study aimed to evaluate the effects of Bu in a mouse model of IBD. Oral administration of Bu increased beneficial commensal bacteria, including *Bacillus* and *Bifidobacterium*, while reducing pro-inflammatory bacteria, such as *Escherichia*-*Shigella*, thereby promoting a diverse and favorable microbial community. Simultaneously, Bu facilitated the metabolic production of more bile acids, which inhibited the differentiation of colonic TH17 cells and significantly slowed the development of IBD. Our study provides insights into the therapeutic use of Bu for the treatment and prevention of IBD.

## Results

### *B. uniformis* JCM5828 ameliorates DSS-induced colitis

**Fig. 1:**
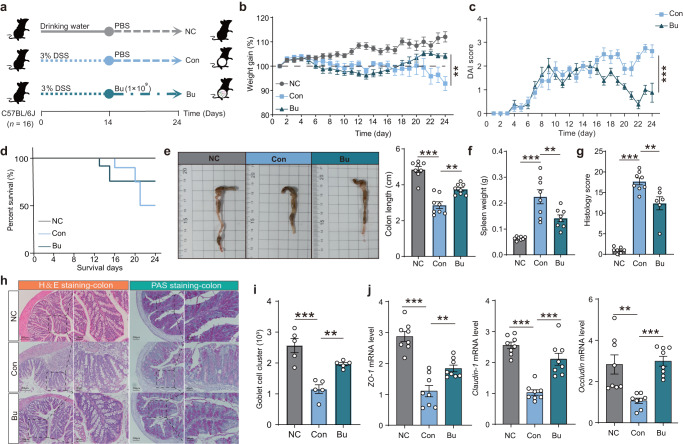
*B. uniformis* JCM5828 ameliorates DSS-induced colitis by protecting against intestinal barrier damage and tight junction disruption. **a** Schematic of the experimental design. Mice (female, *n* = 16 per group) were given 3.0% DSS for 24 d and treated with PBS or *B. uniformis* JCM5828 from d 15 to d 24. The number of mice surviving in the Con group on the day of sampling (*n* = 8) defined the final biological replicate of each group. **b** Changes in body weights during the experiments (*n* = 8). **c** DAI scores during the experiments (*n* = 8). **d** Survival curve during the experiments (*n* = 16). **e** A representative image of colon tissue from each group was provided, and the colon length was recorded (*n* = 8). **f** The spleen weight of each group (*n* = 8). **g** The histological score of the DSS-induced colitis was evaluated. **h** Representative microscopic images of mouse colon tissue stained with H&E and PAS (Scale bars = 200 μm) and corresponding local high magnification images (Scale bars = 50 μm). **i** The goblet cell count of the DSS-induced colitis was evaluated (*n* = 5). **j** qPCR analysis showing the mRNA expression of Occludin, *ZO-1*, Claudin-1 in colon tissues (*n* = 8). **p < 0.01, ***p < 0.001. Data were analyzed using one-way ANOVA with Tukey’s test and expressed as the means ± SEM.

To investigate the potential remission effect of Bu on DSS-induced colitis in mice, DSS-induced C57BL/6 J colitis mice were treated with the Bu strain for 14 d (Fig. 1a). Compared to the Con group, Bu treatment significantly alleviated DSS-induced weight loss (Fig. 1b), decreased the disease activity index (DAI) scores (Fig. 1c), and improved the survival rate (Fig. 1d). Additionally, Bu treatment substantially reduced colon inflammation, as evidenced by longer colon lengths and lower spleen weights (Fig. 1e, f). The cumulative scores of inflammatory infiltrates, goblet cell loss, crypt density, submucosal inflammation, and crypt abscesses were significantly reduced in the Bu-treated group compared to those in the Con group (Fig. 1g, h). Furthermore, Bu treatment significantly increased mucus layer thickness and the number of goblet cells in the colon (Fig. 1h, i), as well as colonic mRNA and protein levels of *ZO-1*, Claudin-1, and Occludin (Fig. 1j and Supplementary Fig. 1a). Conversely, Bu treatment significantly decreased the mRNA levels of the pro-inflammatory cytokines *TNF-α*, *IL-6*, and *IL-1β* (Supplementary Fig. 1b). These results suggest that Bu can effectively restore the colonic mechanical and immune barriers, inhibiting the secretion of pro-inflammatory cytokines, thus alleviating DSS-induced colitis.

### *B. uniformis* JCM5828 reshapes microbial community and alleviates DSS-induced colitis

**Fig. 2:**
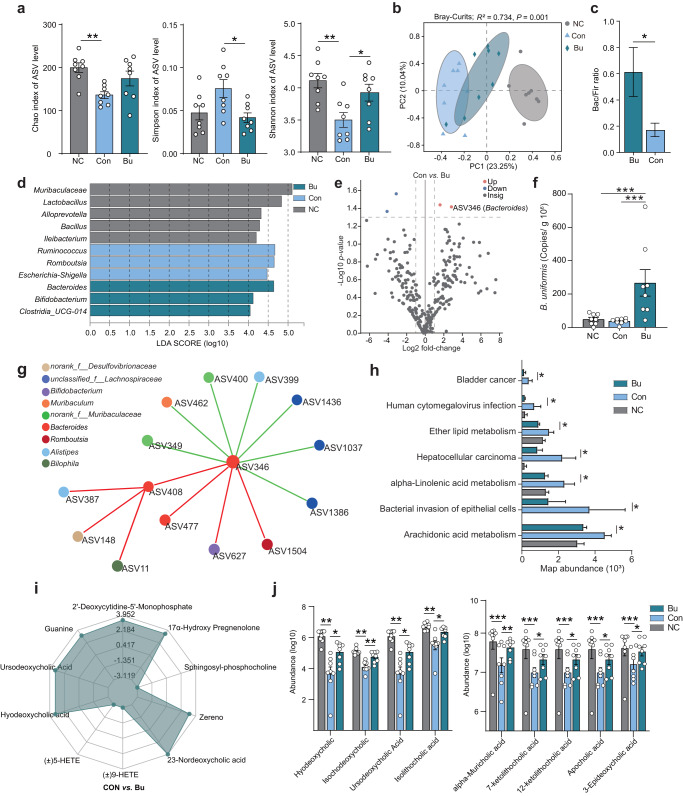
*B. uniformis* JCM5828 reshapes the intestinal microbiota and regulates intestinal bile acid metabolism. Samples of colonic content from mice that were euthanized after treatment or died naturally during treatment were collected for 16 S rRNA and metagenomic sequencing. **a** The α diversity based on Chao, Simpson, and Shannon indexes (female, *n* = 8 per group). Data were analyzed using Kruskal–Wallis with Tukey–Kramer’s test. **b** Principle coordinate analysis (PCoA) plot based on the ASV matrix in three groups. β-diversity was determined using Adonis with Bray–Curtis test. **c** The Bacteroides to Firmicutes (Bac/Fir) ratio in three groups. Data were analyzed using one-way ANOVA with Tukey’s test. **d** Bacterial taxa identified as differentially abundant between groups according to LEfSe. The bacterial taxa of the three groups were compared at the genus level. Data were analyzed using the one-against-all multi-group comparison strategy, with an LDA threshold set at >4.0. **e** Differentially enriched bacteria between Bu and Con groups. Blue dots represent bacteria with higher abundance in the samples of Bu group. Gray dots represent bacteria with no significant difference between the two groups. *P* values were adjusted by Benjamini–Hochberg (BH) method to control FDR. FDR-adjusted *P* < 0.05 was shown. **f** Copy number of Bu in mouse colonic contents. Data were analyzed using one-way ANOVA with Tukey’s test. **g** Colonic microbial co-occurrence network analysis based on core ASV. Spearman’s rank correlation coefficient >0.60; *P* value <0.05. Different colors represent different genera in the colon. The size of nodes is proportional to the relative abundance of the ASV. The red and green lines indicate positive and negative correlations between species, respectively. **h** The potential functional pathways of colonic content microbiota based on PICRUSt2. Data differences were assessed using Kruskal–Wallis with Tukey–Kramer’s test. **i** Radar plot of differential metabolites in Con vs. Bu groups. Grid lines represent log2 values of difference multiples. **j** The relative abundance of nine differential metabolites in three groups. Differential metabolites were defined as metabolites with a fold change ≥2 and ≤0.5. Data were analyzed using Kruskal–Wallis with Tukey–Kramer’s test. *p < 0.05, **p < 0.01, ***p < 0.001. Data were expressed as the means ± SEM.

To further assess the regulation of the colonic microbial community in mice by Bu oral administration, we analyzed the composition and potential functional changes in the colonic microbial community in three groups of mice using 16S rRNA gene sequencing. Compared with the Con group, bacterial commensal richness, and diversity according to Simpson and Shannon indices significantly increased in the Bu group (*P* < 0.05, Fig. 2a). Principal coordinate analysis (PCoA) showed that the three groups had significantly different microbial community compositions at the ASV level (Adonis; Bray–Curtis, *R*^2^ = 0.734, *P* = 0.001; Unweighted-Unifrac, *R*^2^ = 0.774, *P* = 0.001; Abund–Jaccard, *R*^2^ = 0.808, *P* = 0.001; Fig. 2b, Supplementary Fig. 2a, and Supplementary Table 1). Bu treatment significantly increased the Bacteroides to Firmicutes (Bac/Fir) ratio from 0.17 to 0.61 (*P* = 0.01, Fig. 2c). Biomarkers with biological consistency identified using Linear discriminant analysis Effect Size (LEfSe) analysis characterized phenotypic changes in taxonomic composition. IBD biomarker strains, including *Escherichia*, *Shigella*, and *Ruminococcus*, were identified as the core microbes in the Con group, whereas *Bacteroides* and *Bifidobacterium* were identified as the core microbes in the Bu group (LDA >4.0; Fig. 2d). Bu treatment significantly compensated for the lack of ASV85 (*Lactobacillus vaginalis*, *P* < 0.05) and ASV469 (*Alistipes*, *P* = 0.02) abundance and increased the abundance of ASV346 (*Bacteroides*, *P* = 0.02) and ASV622 (*Bifidobacterium*, *P* = 0.02) in the colon of DSS-induced colitis mice (Supplementary Fig. 2b and Supplementary Table 2), especially for ASV346 (*Bacteroides*), which was significantly increased in the Bu group as determined by the Log2 fold-change in Con *vs*. Bu (Fig. 2e and Supplementary Fig. 2c). Bu treatment significantly reduced the abundance of the potentially pathogenic bacteria ASV178 (*Escherichia-Shigella*, *P* < 0.05) and ASV92 (*Klebsiella*, *P* < 0.01) in the colon of DSS-induced colitis mice (Supplementary Fig. 2b and Supplementary Table 2). It is worth noting that the number of copies of Bu in the Bu group significantly increased compared with that of the Con group using qPCR (*P* < 0.01, Fig. 2f). The interaction network of the strains further showed that ASV346 (*Bacteroides*) cooperated with ASV408 (*Bacteroides*), ASV477 (*Bacteroides*), ASV627 (*Bifidobacterium*), and ASV 1504 (*Romboutsia*) to reduce the abundance of ASV462 (*Muribaculum*) and ASV399 (*Alistipes*) (*r* = 0.6, *P* < 0.05; Fig. 2g).

PICRUST2 was used to predict microbial function, and significant differences were identified in 40 characteristic microbiota pathways between the Bu and Con groups (Supplementary Table 3). Moreover, disease pathways, including those related to pathogenic *Escherichia coli* infection, *Salmonella* infection, Huntington’s disease, and *Vibrio cholerae* infection, were significantly downregulated in the Bu group compared with the Con group (Supplementary Fig. 2d). Similarly, many key lipid metabolic pathways, such as those of ether lipid, alpha-linolenic acid, arachidonic acid, and glycerophospholipid, were significantly downregulated in the Bu group when compared with that of the Con group (Fig. 2h). These findings suggest that Bu can reshape the colonic microbial composition of mice with DSS-induced colitis. Additionally, Bu can induce potential synergistic probiotics to suppress the abundance of pathogenic bacteria and modulate the expression of colonic lipid metabolism pathways, thereby alleviating colitis.

### *B. uniformis* JCM5828 alters colonic bile acid profile in mice with DSS-induced colitis

Comparison of the metabolites of the NC vs. Con, NC vs. Bu, and Con vs. Bu groups using OPLS-DA analysis showed satisfactory Q2Y values of 0.911, 0.867, and 0.871, and R2Y values of 0.997, 0.994, and 0.997, respectively, indicating substantial separation of metabolites among the groups (Supplementary Fig. 3a). A total of 219 and 61 differential metabolites (DMs) were identified when comparing NC vs. Con and Con vs. Bu groups, respectively, of which 117 and 52 DMs were upregulated in the NC vs. Con, Con vs. Bu groups, respectively (Supplementary Fig. 3b and Supplementary Tables 4, 5). Calculating the differences between the metabolites resulted in different subgroups, and then the top 10 metabolites with the largest differences were selected for radar plotting (Fig. 2i and Supplementary Fig. 3c). The DSS treatment considerably reduced the concentration of ursodeoxycholic (UDCA) and hyodeoxycholic acid (HDCA) in the mice colon (Supplementary Fig. 3c, d), whereas Bu treatment substantially restored UDCA and HDCA concentrations (Fig. 2i and Supplementary Fig. 3d). Comparing the relative levels of differential bile acids between Con and Bu groups indicated that Bu treatment increased the abundance of bile acids in DSS-treated mice colon, including isolithocholic (isoLCA), isochodeoxycholic (isoCDCA), alpha-muricholic (α-MCA), apocholic, 12-ketolithocholic, and 7-ketolithocholic acid (Fig. 2j and Supplementary Table 4). These results indicate that the genome of *B. uniformis* JCM5828 contains genes that can regulate bile acid metabolism.

### Genomic basis of *B. uniformis* JCM5828-induced bile acid salt changes

The bile salt tolerance of Bu was determined by examining the number of single-cell clones of Bu strains in media with different bile salt concentrations. The Bu strain survived up to 76.79% when 0.5% bile salt was added to the medium (Supplementary Fig. 4a). To further evaluate the key functional genes contained in Bu, we performed whole genome sequencing of the *B. uniformis* JCM5828 and found that the genome size is 4,660,774 bp, with 46.52% GC content, and the total number of genes were annotated to 1661 using Kyoto Encyclopedia of Genes and Genomes (KEGG). Functional annotation using KEGG revealed that Bu could encode critical enzymes involved in primary bile acid biosynthesis in the lipid metabolism pathway (Supplementary Fig. 4b). On the Bu chromosome, we found via an annotation that gene 2119 encodes choloylglycine hydrolase (*BSH*, EC:3.5.1.24) (Supplementary Fig. 4c). This result confirmed that the genome of the *B. uniformis* JCM5828 strain contains the key enzyme gene *BSH* involved in the hydrolysis of conjugated bile acids (CBA) to unconjugated bile acids (UCBA) and glycine or taurine.

### *B. uniformis* JCM5828 alters colonic transcriptome profile in mice with DSS-induced colitis

**Fig. 3:**
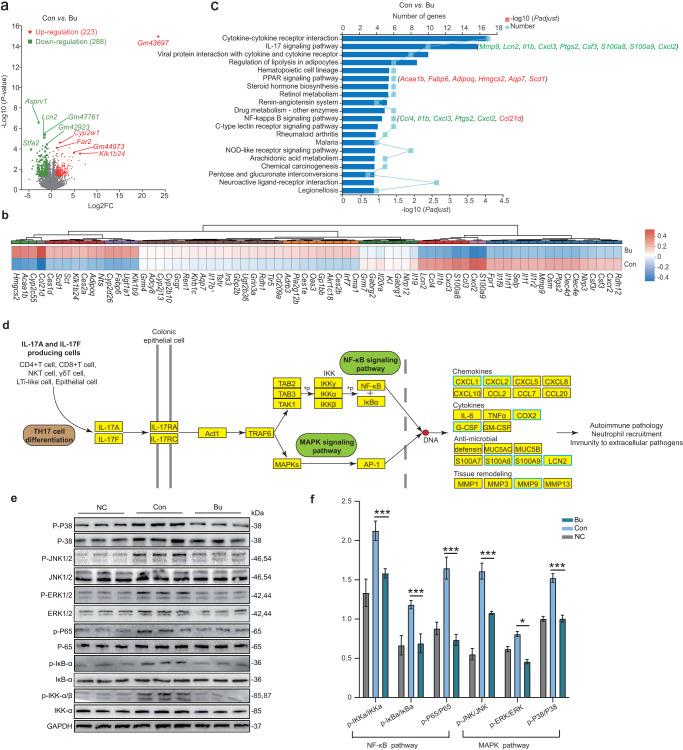
*B. uniformis* JCM5828 alters colonic transcriptome profile in mice with DSS-induced colitis. **a** Volcano plots for the RNA-Seq analyses of Con vs. Bu group. The red and green dots represent upregulated and downregulated DEGs in the Bu group, respectively. **b** The heatmap of DEGs expression in two groups. **c** The KEGG enrichment analysis of Con vs. Bu group. The genes behind each bar represent the gene involved in the pathway. The red and green genes represent upregulated and downregulated DEGs in the Bu group, respectively. **d** Bu binds to the IL-17 receptor (IL-17R) in colon epithelial cells and inhibits downstream NF-κB and MAPK signaling pathways through the signaling complex IL-17R-ACT1-TRAF6, thereby eliminating the transcription and expression of inflammatory factors in the nucleus. The blue box represents KEGG annotated differential genes downregulated in the Bu group. **e**, **f** Western blot analyses of p-P38/P38, p-JNK1/2/JNK1/2, p-ERK1/2/ERK1/2, p-P65/P65, p-IκB/IκB, and p-IKK/IKK in colon tissues using the respective anti-phospho-protein antibodies, and quantified phosphorylated protein and total protein density (*n* = 8). Data were analyzed using one-way ANOVA with Tukey’s test and expressed as the means ± SEM. *p < 0.05, ***p < 0.001.

A total of 143.29 Gb of data were obtained using 19 colonic epithelial samples from the three groups using RNA-Seq. Comparing the NC *vs*. Con and Con *vs*. Bu groups, a total of 2578 and 511 differentially expressed genes (DEGs) were identified, respectively, of which 223 DEGs were upregulated and 288 DEGs were downregulated in the Bu group compared with the Con group (Fig. 3a and Supplementary Fig. 5a). Typical inflammatory chemokines, including *Ccl4*, *Ccl3*, *Cxcl2*, *Cxcl3*, and *Cxcr2*, induces immune cells to enter the site of infection during the immune response. Furthermore, *S100A8* and *S100A9* can activate inflammatory cells through neutrophil chemotaxis and are strongly associated with various tumor diseases. In this study, the expression of *Ccl4*, *Ccl3*, *Cxcl2*, *Cxcl3, Cxcr2, S100A8*, and *S100A9* was largely decreased in the Bu group compared to that in the Con group. In addition, the expression of *Ptgs2*, *MMp9*, and the interleukin-1 family *Il1b*, *Il1r2*, *Il1rl1*, and *Il1f9* was considerably decreased in the Bu group compared to that in the Con group (Fig. 3b and Supplementary Table 6). In contrast, the expression of genes associated with lipid metabolism in the peroxisome proliferators-activated receptor (PPAR) signaling pathway, including ketogenic *Hmgcs2*, adipogenic *Scd1*, *Acaa1b*, *Adipoq*, and *Fabp6*, were noticeably increased in the Bu group compared to that in the Con group. These genes can affect fatty acid oxidation, adipocyte differentiation, and fatty acid binding (Fig. 3b and Supplementary Table 6). KEGG pathway analysis showed that most DEGs (*Mmp9*, *Lcn2*, *Il1b*, *Cxcl3*, *Ptgs2*, *Csf3*, *S100a8*, *S100a9*, and *Cxcl2*) were primarily involved in intracellular inflammatory signaling pathways, including the IL-17 and NF-κB signaling pathways (Fig. 3c). DSS treatment enhanced the activity of the IL-17 signaling pathway in the colon epithelium of mice, but it was inhibited by Bu intervention (Fig. 3c and Supplementary Fig. 5b). RNA-Seq data highlighted that the IL-17 and NF-κB signaling pathways were the most important inflammatory signaling pathways in the Bu group. The IL-17 receptor (IL-17R) can activate downstream NF-κB, MAPK, and other signaling pathways via the signaling complex IL-17R-Act1-TRAF6^21^ (Fig. 3d). Western blot analysis of the key proteins of the NF-κB and MAPK signaling pathways in mouse colon tissue revealed that the ratios of p-NF-κB/NF-κB, p-IκB/IκB, and p-IKK/IKK were significantly decreased in the Bu group (*P* < 0.001, Fig. 3e, f). Consistently, the MAPK pathway was significantly decreased in terms of extracellular signals related to kinases (ERK1/2), Jun amino-terminal kinases (JNK1/2), and p38 of the MAPK protein family (*P* < 0.05, Fig. 3e, f). These results suggest that Bu ameliorates DSS-induced colitis development by inhibiting the IL-17 signaling pathway.

### *B. uniformis* JCM5828-mediated α-MCA, HDCA, and isoLCA inhibits TH17 differentiation in vitro

**Fig. 4:**
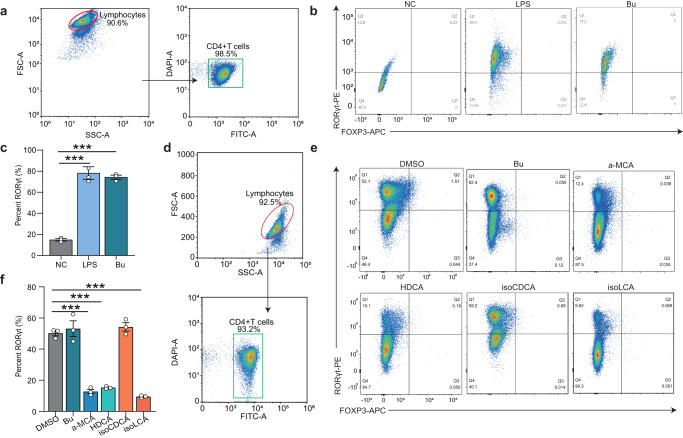
*B. uniformis* JCM5828-mediated bile acids inhibit TH17 differentiation and alleviate DSS-induced colitis. **a** Gating strategy for the flow cytometric sorting of intestinal CD4^+^ T cells. Flow cytometric analyses (**b**) and quantification (**c**) of RORγt production from C57BL/6J mice naive T cells cultured for 3 d under the TH17 cell polarization condition. PBS, PBS, or Bu supernatant (MOI = 1:10) were added after LPS addition (*n* = 3 respectively, biologically independent samples). **d** Gating strategy for the flow cytometric sorting of intestinal CD4^+^ T cells. Flow cytometric analyses (**e**) and quantification (**f**) of RORγt production from C57BL/6J mice naive T cells cultured for 3 d under the TH17 cell polarization condition. DMSO, Bu supernatant (MOI = 1:10), or bile acids (20 μM) were added after cytokine addition (*n* = 3 respectively, biologically independent samples). Data were analyzed using one-way ANOVA with Tukey’s test and expressed as the means ± SEM. ***p < 0.001.

Based on the RNA-Seq data, we hypothesized that Bu could mediate the immune effect of bile acids by inhibiting TH17 cell differentiation. To examine this hypothesis, immunohistochemical staining of TH17 cells in mouse colonic tissue was performed. Comparison with the Con group, showed that the expression of IL-17A in the lamina propria of the colon was significantly reduced in the Bu group, indicating that Bu might inhibit the differentiation of TH17 cells in the colon. (*P* < 0.001, Supplementary Fig. 6). Subsequently, naive CD4^+^ T cells were isolated from lymphocytes in the lamina propria of wild-type C57BL/6J mice (Fig. 4a). Lipopolysaccharide (LPS) was added to the LPS and Bu groups to induce TH17 cell differentiation in vitro, and equal volumes of phosphate buffer saline (PBS) and Bu supernatant were added to co-culture for 3 d. Notably, Bu did not inhibit the differentiation of TH17 cells (*P* > 0.05, Fig. 4b, c), whereas the addition of α-MCA, HDCA, and isoLCA bile acids to lymphocytes significantly inhibited the differentiation of TH17 cells, but isoCDCA addition had no effect in vitro (*P* < 0.001, Fig. 4d–f). These results confirmed that Bu mediates the inhibition of TH17 differentiation by α-MCA, HDCA, and isoLCA in vitro to modulate the colonic immune response. Therefore, a mixture of α-MCA, HDCA, and isoLCA may be evaluated as a potential drug for treating colitis.

### Oral administration of bile acids mixture alleviated DSS‑induced colitis by inhibiting TH17 differentiation in vivo

**Fig. 5:**
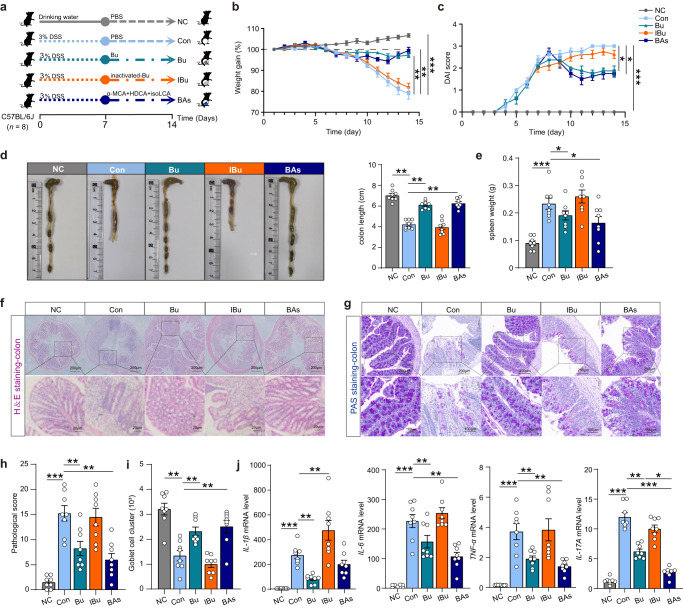
The mixed bile acids attenuated symptoms in the DSS-induced colitis mouse model. **a** Schematic of the experimental design. Mice (female, *n* = 8 per group) were given 3.0% DSS for 14 d and treated with PBS, Bu, inactivated Bu, or mixed bile acids from d 7 to d 14. **b** Changes in body weights during the experiments. **c** DAI scores during the experiments. **d** A representative image of colon tissue from each group was provided, and the colon length was recorded (*n* = 8). **e** The spleen weight of each group (*n* = 8). **f** Representative microscopic image of H&E staining of mouse colon tissue (Scale bars = 200 μm) and corresponding local high magnification images (Scale bars = 20 μm). **g** Representative microscopic image of PAS staining of mouse colon tissue (Scale bars = 200 μm) and corresponding local high magnification images (Scale bars = 100 μm). Histological scores (**h**) and goblet cell count (**i**) of the DSS-induced colitis were evaluated (*n* = 8). **j** qPCR analysis showing the mRNA expression of *IL-1β*, *IL-6*, *TNF-α*, and *IL17A* in colon tissues (*n* = 8). Data were analyzed using one-way ANOVA with Tukey’s test and expressed as the means ± SEM. *p < 0.05, **p < 0.01, ***p < 0.001.

To further verify the effect of mixed bile acids on colitis, Bu, inactivated Bu (IBu), and mixed bile acids (α-MCA, HDCA, and isoLCA, mixed in equal proportions) were administered for 7 d to a DSS-induced colitis mouse model, which consisted of negative control (NC) group, DSS (Con) group, Bu group, IBu group, and mixed bile acids (BAs) group (Fig. 5a). Both Bu and BAs treatments alleviated DSS-induced weight loss and reduced DAI scores compared to the Con group, whereas IBu had no effect (Fig. 5b, c). In addition, Bu and BAs treatment restored colonic length and spleen weight in mice compared to the Con group, whereas, again, IBu treatment had no effect (Fig. 5d, e). Bu and BAs treatment markedly reduced cumulative scores characterized by inflammatory infiltration, goblet cell loss, crypt density, submucosal inflammation, and crypt abscesses, and substantially increased mucus layer thickness and goblet cell numbers (Fig. 5f–i), as well as mRNA and protein levels of *ZO-1*, Claudin-1, and Occludin in the colon, when compared to the results of the Con group (Supplementary Fig. 7). Similarly, the intestinal mechanical barrier in the IBu group was consistent with that in the Con group. In addition, consistent with the results of Bu treatment, BAs treatment significantly reduced the mRNA levels of *TNF-α*, *IL-6*, and *IL-17A* in mice colon when compared with the mRNA levels of the Con group (Fig. 5j). These results suggest that both Bu and mixed bile acids can effectively restore the mechanical and immune barriers of the colon and inhibit the secretion of pro-inflammatory cytokines, thereby alleviating DSS-induced colitis.

**Fig. 6:**
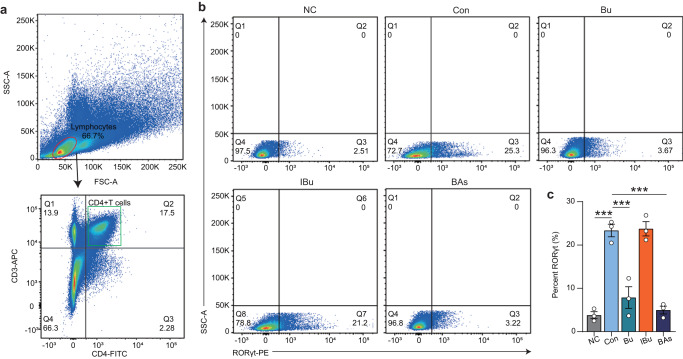
The mixed bile acids inhibits TH17 differentiation. **a** Gating strategy for the flow cytometric sorting of intestinal CD4^+^ T cells. Flow cytometric analyses (**b**) and quantification (**c**) of Th17 (CD3^+^CD4^+^RORγt^+^) frequencies in the colonic lamina propria of C57BL/6J mice in each group (*n* = 3 respectively, biologically independent samples). Data were analyzed using one-way ANOVA with Tukey’s test and expressed as the means ± SEM. ***p < 0.001.

Next, we investigated whether Bu and mixed bile acid gavage inhibited the differentiation of mouse colonic TH17 cells. TH17 cells (CD4^+^CD3^+^RORt^+^) were identified from the lamina propria lymphocytes of each group of mice (Fig. 6a, b). Notably, Bu and BAs treatment significantly inhibited the differentiation of TH17 cells when compared with the Con group results, whereas IBu failed to regulate the differentiation of TH17 cells (Fig. 6c). Overall, these results suggest that Bu can regulate αMCA, HDCA, and isoLCA concentrations in the colon, inhibiting TH17 differentiation in colonic epithelial cells and the expression of downstream NF-κB and MAPK signaling pathways, thus alleviating DSS-induced colitis.

## Discussion

**Fig. 7:**
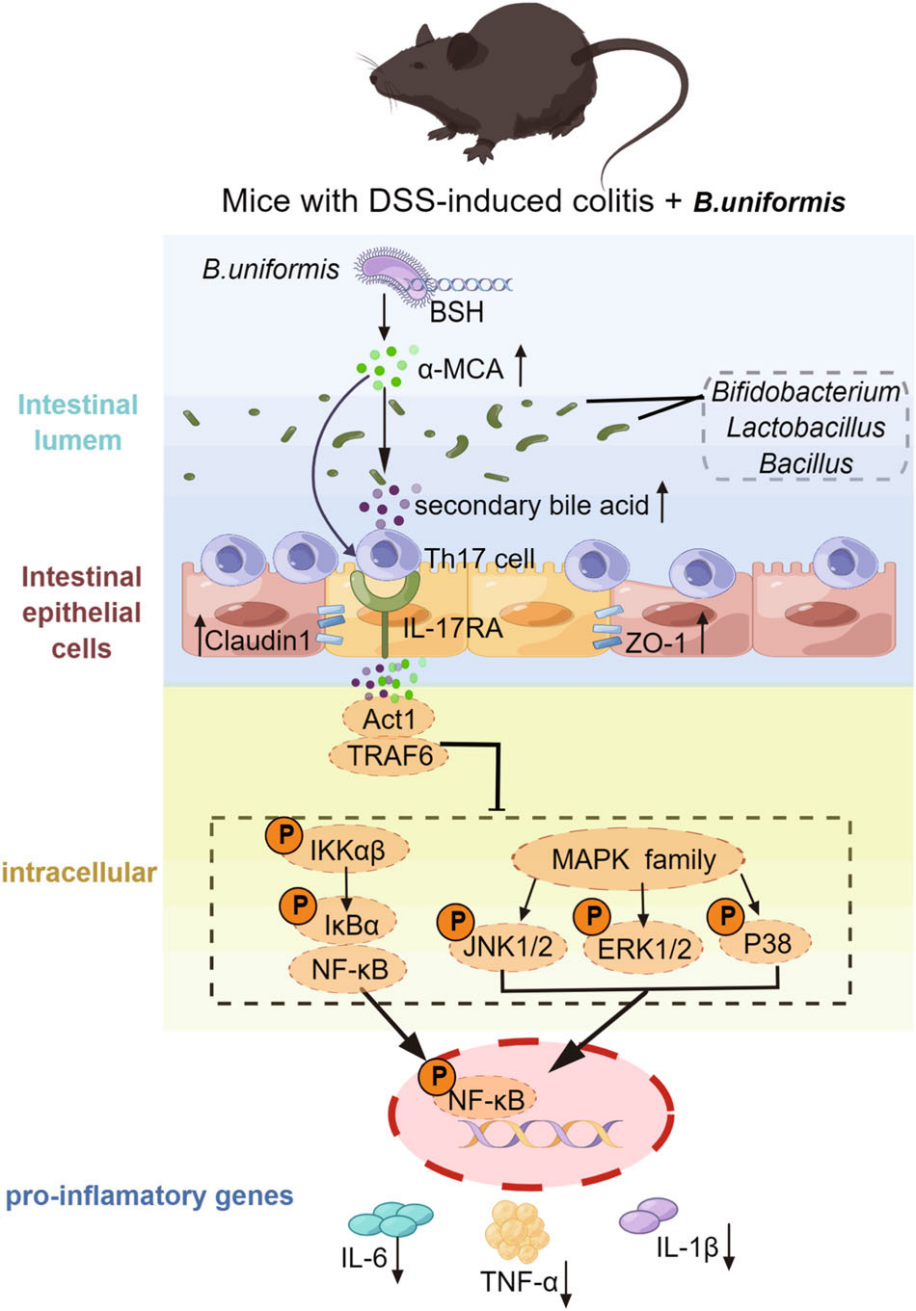
The processes behind the protective effect of *B. uniformis* JCM5828 against DSS-induced colonic inflammation in mice are summarized in a schematic picture. Bu gavage changed the colonic microbial composition of mice with DSS-induced colitis, and primary bile acids (α-MCA) synthesized by the Bu core functional enzyme gene BSH involved in enhancing intestinal barrier function and inhibiting TH17 cell differentiation along with other putative probiotic-mediated secondary bile acids. TH17 cells in the colon epithelium bind to the IL-17 receptor (IL-17R) and inhibit downstream NF-κB and MAPK signaling pathways via the signaling complex IL-17R-ACT1-TRAF6. This reduces the expression of inflammatory factors such as TNF-α, IL-1β, and IL-6, modulating the intestinal immune response. Figdraw software was used to create this illustration.

Previous studies have shown that long-term treatment of mice with *B. uniformis* CECT 7771 can alter the gut microbial composition and promote the proliferation of potentially beneficial bacteria^22,23^. *B. uniformis* CECT 7771 can enhance the activity of macrophages and dendritic cells (DCs), restore the ability of DCs to recognize antigens, and stimulate the proliferation of T lymphocytes^22^. *B. uniformis* CECT 7771 can also reduce serum cholesterol, triglyceride, glucose, and insulin levels in obese mice, and the number of fat particles in the small intestine, thus alleviating the metabolic and immune disorders caused by high-fat diets^24^. DSS induces colon epithelial damage and promotes the proliferation of harmful bacteria, causing diarrheal phenotypes in animals^25^. Several studies have shown that Th17 cells are involved in the pathogenesis of DSS-induced colitis^26,27^. In this study, Bu altered DSS-induced colonic microbial composition and bile acid profile, especially key metabolites (αMCA, HDCA, and isoLCA), which, as immune signaling molecules, alleviated colonic inflammation by inhibiting the differentiation of TH17 cells (Fig. 7). The mucus layer thickness can be used to assess the function of the intestinal barrier and protect the epithelium from harmful factors^28^. The tight junction proteins can effectively prevent the paracellular transport of bacteria, toxins, and other substances into the intestinal lumen through regulation, thus maintaining the integrity of the epithelial barrier function of the intestinal mucosa^29,30^. In addition, pro-inflammatory cytokines, such as *IL-6*, *TNF*, and *IL-1β*, play an important role in regulating the intestinal inflammatory response. Inflammation promotes the development and progression of colorectal cancer in the inflammatory microenvironment by silencing tumor suppressors, causing damage to cellular DNA, and upregulating anti-apoptotic protein genes through the release of large amounts of pro-inflammatory cytokines from activated T cells and macrophages^31,32^. According to the present study, both Bu and mixed bile acids could alleviate the DSS-induced impairment of the colonic mucosal immune barrier and inhibit the expression of pro-inflammatory cytokine genes, thus triggering the Th17 response.

Previous studies have demonstrated that IBD development is associated with gut microbiota^33,34^. Some functional strains used for IBD treatment rely on probiotics. For example, the *Gassericin A* bacteriocins secreted by *L. gasseri* LA39 and *L. frumenti* can upregulate intestinal fluid absorption-related proteins and improve intestinal epithelial barrier function in early weaned piglets by interacting with the host KRT19 protein^35^. *Akkermansia muciniphila* secretes a P9 protein that induces the secretion of glucagon-like peptide-1, thus ameliorating glucose homeostasis and metabolic diseases in mice^36^. However, most strains exert probiotic effects by changing the gut microbial composition and regulating host metabolism. For example, the microbiota-associated metabolites taurine, histamine, and spermine can ameliorate colitis via synergistic regulation of NLRP6 inflammasome signaling, epithelial IL-18 secretion, and downstream antimicrobial peptide profiles, thus restoring normal microbiota^37^. In this study, Bu exerted anti-inflammatory effects in the intestine mainly by synergizing with other gut strains, including *Bifidobacterium*, *Bacteroides*, and *Lactobacillus*, thus altering the colonic bile acid profile and suppressing the abundance of *Ruminococcus* and *Escherichia-Shigella* pathogenic bacteria. Previous studies have shown that some *Bifidobacteria* and *Lactobacillus* have anti-inflammatory functions. For instance, *Bifidobacterium pseudolongum* can improve the human immune response, prevent intestinal diseases, inhibit allergies, and lower cholesterol^38^. *Lactobacillus vaginalis* can produce various bacteriocins, such as acidolin, acidophilin, and laetocidon, thus preventing the growth of pathogenic microorganisms due to their good antibacterial effect^39^. *Ruminococcus-gnavus* secretes glucorhamnan, a complex polysaccharide with a rhamnose skeleton and glucose side chain, which induces dendritic cells to secrete inflammatory cytokines (TNF-α), thus leading to colitis development^40^. Excess *Ruminococcus* is a key indicator of irritable bowel syndrome^41^. *Escherichia-Shigella* is an adherent-invasive bacterium whose abundance increases in patients with UC, thus aggravating colitis development^42^.

Genomic sequencing has also shown that the Bu gene encoding BSH is involved in UCBA biosynthesis in the host colon^43^. Furthermore, both sterile and antibiotic-treated mice showed substantially increased CBA accumulation in the intestinal cavity and largely decreased levels of secondary bile acids^44^. In addition, colonized BSH knockout *B. thetaiotaomicron* considerably affected bile acid metabolism in sterile mice^45^. These results further suggest that *BSH* may be involved in the biosynthesis of primary bile acids (α-MCA). However, 7α-hydroxy steroid dehydrogenase, which plays a crucial role in the conversion of αMCA into secondary bile acids such as HDCA, was not identified via annotation on the Bu chromosome^46–48^. Therefore, Bu regulates the biosynthesis of secondary bile acids by regulating the structure of the gut microbiota through other functional strains. Nevertheless, host enzymes may be involved in this process. It has been shown that bicyclol reduces liver injury in mice by increasing the levels of α-MCA, regulating bile acid metabolism, and improving histopathological parameters^49^. However, it is unknown whether α-MCA can alleviate inflammation through TH17 cells. HDCA can improve the intestinal bile acid profile and induce anti-inflammatory effects in mice by inhibiting intestinal pro-inflammatory factor production in the bile acid receptor (FXR)^50^. IsoLCA inhibits TH17 differentiation by binding to RORγt and inhibiting its transcriptional activity. Furthermore, colonization of mice with enterobacteria-producing isoLCA decreases the number of TH17 cells^10^ and the secretion of pro-inflammatory cytokines, exacerbating pathogen invasion and decreasing host immunity^51,52^. However, our study shows that Bu synergizes with intestinal microbiota to regulate the biosynthesis of α-MCA, HDCA, and isoLCA in mice, and that these bile acid metabolites modulate intestinal immune response by inhibiting the differentiation of intestinal TH17 cells. Therefore, metabolites from the gut microbiota can mediate complex interactions between the gut microbiota and the host.

IL-17 and NF-κB comprise the most important inflammatory signaling pathways^53,54^. The interaction between both pathways amplifies inflammatory signals via the MAPK pathway. Receptor proteins in the inflammatory pathway can regulate gene transcription and expression by binding to the promoter and enhancer sequence sites of various pro-inflammatory genes, exacerbating the inflammatory response^55,56^. NF-κB dimers are sequestered in the cytosol of unstimulated cells via interactions with their inhibitor, IκB. IκB can be phosphorylated after lipopolysaccharide stimulation, leading to the release of NF-κB. Then, phosphorylated NF-κB translocates to the nucleus and activates cytokine expression^55^. The MAPK signaling pathway is shared by four distinct cascades (ERK1/2, JNK1/2/3, p38-MAPK, and ERK5)^57,58^. However, the role of ERK in inflammation remains controversial. Some studies have found that decreased ERK can suppress inflammation^59,60^, whereas other studies have reported that ERK activation can protect against inflammation^61^. Our results demonstrate that Bu majorly suppresses the phosphorylation of IκK (IκB kinase), IκB, NF-κB, ERK1/2, JNK1/2, and p38-MAPK, thus relieving inflammation. Therefore, Bu alleviates colitis by regulating bile acid metabolism in the host and inhibiting IL-17 signaling pathway activity and TH17 cell differentiation.

In this study, gut microbe-metabolites were found to control host immune responses by directly modulating immune cells. TH17 cells play critical roles in various inflammatory diseases and are closely associated with gut bacteria. Novel modulatory pathways that regulate T cell function through microbe-mediated bile acid metabolites were identified. Bile acids can regulate host physiology and immune responses, indicating that understanding the role of host-microbiota networks in mediating bile acid biotransformation can provide therapeutic interventions for IBD. Therefore, future studies should elucidate the Bu or host enzymes that generate bile acids for the effective control of T cell function in the context of autoimmune diseases and other inflammatory conditions.

## Methods

### Isolation of *B. uniformis* JCM5828 using improved Brucella Broth medium

The *B. uniformis* JCM5828 strain was isolated from the feces of 60-day-old goats. Briefly, the feces were transferred to a 5 mL sterile cryopreservation tube containing 30% glycerol immediately after collection and stored at −80 °C after snap-freezing in liquid nitrogen. Each 1 g of feces per sample was suspended in PBS buffer and diluted by a gradient up to 10^−8^. Then, 0.01‰ heme chloride (16009-13-5, Yuanye, China), 0.01‰ vitamin K1 (84-80-0, Sangon, China), 0.1‰ kanamycin (8063-07-8, bidepharm, China), 0.0075‰ vancomycin (1404-90-6, Macklin, China), and 5% defibrinated sheep blood (TX0030, Solarbio, China) were added to the original Brucella Broth medium (LA3560, Solarbio, China) forming improved Brucella Broth medium. The sample (50 μL) was used to coat the solid medium, then it was cultured anaerobically at 37 °C for 48 h. Single colonies were then selected for species identification, and target strains were obtained.

### DNA extraction and bacteria quantification

A TIANamp Bacterial DNA Kit (DP302-02, Tiangen, China) was used to extract genomic DNA from *B. uniformis* JCM5828. The 16S rDNA was amplified via PCR (TransGen, China, 2×EasyTaq PCR SuperMix) using the following reaction primers: forward primer: 5′- AGAGTTTGATCCTGGCTCAG-3′, reverse primer: 5′-TACGGYTACCTTGTTACGACTT-3′. The isolated DNA samples were diluted and used as PCR templates. The amplified PCR products were subjected to agarose gel electrophoresis, and the isolated PCR products were sent to AuGCT DNA-SYN Biotechnology Co., Ltd. (Beijing, China) for sequencing.

### Preparation of *B. uniformis* JCM5828

Purified *B. uniformis* JCM5828 was cultured anaerobically at 37 °C for 24 h, then the cultured liquid (50 mL) was centrifuged at 12,000 × *g* and 4 °C for 10 min. The supernatant was discarded, and the sample was washed with PBS and suspended in normal saline for gradient dilution to adjust the concentration of *B. uniformis* JCM5828 for subsequent intragastric treatment.

### Experimental animals

A total of 88 female C57BL/6J mice of 8 weeks of age were purchased from Chongqing Tengxin Biotechnology Co. Ltd. (Xi’an, China). The mice were kept in the Animal Experiment Center of Northwest A&F University under SPF conditions, ambient temperature 23 ± 1 °C, humidity 55 ± 5%, and pathogen-free conditions on a 12/12 h light/dark cycle with free access to food and water. The diet composition and nutritional value are shown in Supplementary Table 7. The experiment was approved by the Institutional Animal Care and Use Committee of Northwest A&F University (permit numbers: 2021-06-008 and 2023-02-010).

### Colitis model and treatments

Experiment 1: Three groups: negative control (NC), positive control (Con), and *B. uniformis* group (Bu). Mice were treated with DSS consecutively for 24 d, except for the NC group, and gavage treatment was given once a day on d 14 and continued for 10 d.

Experiment 2: Five groups: negative control (NC), positive control (Con), *B. uniformis* group (Bu), inactivated *B. uniformis* group (IBu), and mixed bile acids (BAs) group. However, 24 d of DSS treatment resulted in a high mortality rate in mice. Therefore, mice, except for the NC group, were treated with DSS consecutively for 14 d, and gavage treatment once a day starting on d 7 and continued for 7 d.

Throughout the experiment, mice in the NC group were given sterile water, and mice in the remaining groups were given 3% (w/v) DSS (MP Biomedicals, Santa Ana, CA, USA; molecular weight: 36,000-50,000) in sterile water to establish the mouse model of colitis. Mice in the NC and Con groups (DSS + PBS) were gavaged with sterile PBS (200 μL) as negative and positive controls; Bu (DSS + Bu) and IBu (DSS + IBu) groups were gavaged with active and inactive *B. uniformis* JCM5828 (200 μL of 1 × 10^9^ colony forming unites (CFU), respectively); and BAs group (DSS + BAs) were gavaged with a bile acid mixture (200 μL) consisting of three bile acids (αMCA, HDCA, and isoLCA) mixed in equal proportions (50 mg/kg body weight) and then sterilized by filtration through a 0.22 mm filter (SLGV033RB, Millipore Corporation, USA).

Body weight, food intake, and diarrhea scores were recorded each day. The DAI score was determined according to standard procedure^62^. Rectal bleeding scores were as follows: 0 = normal colored stool, 1 = brown stool, 2 = reddish stool, and 3 = bloody stool; diarrhea: 0 = well-formed stools, 1 = mildly soft stool, 2 = very soft stool, and 3 = watery stools.

### Tissue collection, fixation, and histochemistry

The colon samples were fixed in 4% polyformaldehyde, embedded in paraffin, sectioned, and stained with hematoxylin and eosin (H&E) for pathological analysis. Two independent investigators, blinded to the treatment, evaluated the slides. The inflammatory infiltrate, goblet cell loss, crypt density, muscle thickening, submucosal inflammation, crypt abscess, and ulcerations were described using a 0-4 point scale according to standard procedure^63^. Immunohistochemistry was performed using the primary antibodies of rabbit anti-IL-17A (1:100, eBio17B7, eBioscience^TM^, USA), visualized using 3,3′-diaminobenzidine, and counterstained using hematoxylin. Image J software (National Institutes of Health, Bethesda, MD, USA) was used to determine cell numbers.

### RNA extraction and quantitative reverse transcriptase PCR

TRIzol Reagent (Cwbio, China) was used to extract RNA from colon tissues. The total RNA was then reverse-transcribed using the RevertAid First Strand cDNA Synthesis Kit (Thermo Fisher Scientific, USA) to obtain cDNA. qRT-PCR was performed using ChamQ Universal SYBR qPCR Master Mix (Vazyme, China) in the Light Cycler®96 Real-Time PCR System (Roche, USA). All primers used in this study were designed and preliminarily verified using Oligo 7 software and Primer-BLAST (https://www.ncbi.nlm.nih.gov/tools/primer-blast/). The primers were synthesized by Zhongke Yutong (Xi Bostan, China) company (Supplementary Table 8). The comparative cycle method (2 − ΔΔCt) was used to determine relative mRNA expression.

### DNA isolation and bacterial absolute quantification

The Stool DNA Kit (D4015, Omega Biotek, USA) was used to extract DNA from 24 colonic content samples (*n* = 8) following the kit’s operating instructions. A Nanodrop 2000 UV-VI spectrophotometer (Thermo Fisher Scientific, Wilmington, DE, USA) was used to determine the concentration and purity of the extracted DNA, and 1% agarose gel electrophoresis was conducted to assess the DNA quality. The extracted colonic contents DNA was used as a template for PCR amplification using the Bu-specific primers 338 F (5′-TCTTCCGCATGGTAGAACTATTA-3′) and 806 R (5′-ACCGTGTCTCAGTTCCAATGTG-3′)^64^. After recovering the amplification product, the target fragment was ligated into the *pEASY*_T1 Cloning Vector (EC0112, TransGen, China) to construct a standard plasmid and transformed into DH5α competent cells (CT101-01, Thermo Fisher Scientific, USA) in expanded culture. The PCR products were sent to AuGCT DNA-SYN Biotechnology Ltd. for sequencing. The results were compared using the NCBI tool BLAST (www.ncbi.nih.gov/BLAST). The standard plasmids were extracted using the Plasmid kit (DP105, Tiangen, China), and the standard curve was plotted at a 10-fold concentration gradient dilution. Bu-specific primers were used for qPCR amplification, and the bacterial copy number was calculated based on the Ct value.

### 16S rRNA sequencing

The V3-V4 16S rDNA region of colonic contents DNA was amplified using the primers 338F (5′-ACTCCTACGGGAGGCAGCAG-3′) and 806 R (5′-GGACTACHVGGGTWTCTAAT-3′), and then the purified amplicons were combined into equimolar ratios for up-sequencing. Sequencing was performed by Majorbio BioTech Co. (Shanghai, China). The analysis is described online at the Majorbio Cloud Platform (http://cloud.majorbio.com)^65^. Briefly, the sequences were multiplexed using FLASH 1.2.7, and then double-ended reads were merged using FASTP 0.19.6^66^. The sequences were then filtered, denoised, merged, and chimeras removed using the DADA2 plugin in the QIIME2 platform (https://qiime2.org/)^67^. The number of reads per sample was pumped flat to 23,400 to minimize the effect of sequencing depth on the alpha and beta diversity analyses. Finally, the data achieved an average Good’s coverage of 99.83%. Amplicon sequence variants (ASVs) were assigned for classification using the naive Bayes consensus taxonomy classifier for SILVA 138/16s_bacteria in QIIME2.

### RNA-seq analysis

Extraction of total RNA from colon tissue samples and determination of the concentration and purity of the extracted RNA was performed using Nanodrop 2000. RNA-Seq was performed by Majorbio BioTech Co. (Shanghai, China), Shanghai, China. The Illumina Novaseq 6000 platform was used to construct RNA libraries and generate reads of 300 bp long paired-end (Illumina, San Diego, CA, USA). The read number of each gene was transformed into FPKM (fragments per kilobase of exon model per million mapped reads). After quality control of the raw data, the clean data were compared with the house mouse reference genome (GCF000001635.27) using TopHat software to obtain mapped data, which were used for transcript assembly and expression calculation. The expression levels of genes and transcripts were quantified using RSEM (http://deweylab.github.io/RSEM/). After obtaining read counts of genes, differentially expressed genes were identified using the DESeq2 (V1.24.0) package^68^, and the identification criteria were *P adjust* <0.05 and |log2FC|≥ 1. KEGG pathway enrichment analysis of the DEGs was performed using KOBAS 2.0 (http://kobas.cbi.pku.edu.cn/home.do)^69^, and to control the occurrence of false positives in the calculation process, the Bonferroni-corrected for multiple testing correction, and the corrected *P* < 0.05 defaulted for significantly different pathways.

### Immunofluorescence staining

Paraffin-embedded tissue sections were stained using ZO-1 antibody (1:100, ab221547, Abcam, UK), Claudin-1 antibody (1:1000, ab211737, Abcam, UK), and DAPI (C0060, Solarbio, China) for immunofluorescence. Two investigators, blinded to the treatment, independently evaluated the slides. SlideViewer 2.5.0 (DHISTECH Ltd., Hungary) was used for imaging, and Image J software (National Institutes of Health, Bethesda, Maryland) was used to analyze the fluorescence signal intensity.

### Western blot analysis

Colon tissues were weighed and homogenized in cell lysis buffer (Beyotime, China). The homogenate was then centrifuged at 15,000 × *g* and 4 °C for 15 min to obtain the supernatant. Omni-Easy™ instant BCA protein assay kits (Epizyme, China) were used to quantify the protein concentration following the manufacturer’s instructions. The proteins were separated using 10% SDS polyacrylamide gel and then transferred onto PVDF membranes. The membranes were blocked with 5% skimmed milk for 1 h, then immunoblotted with primary antibodies against IKK-α (1:1000, ab32041, Abcam, UK), Phospho-IKKα/β (Ser176/180) (1:1000, #2697, CST, USA), IKB-α (1:1000, ab32518, Abcam, UK), IKB-α (phospho S36) (1:10000, ab133462, Abcam, UK), NF-κB p65 (1:1000, #8242, CST, USA), NF-κB p65 (phospho Ser536) (1:1000, ab76302, Abcam, UK), p38-MAPK (1:1000, #8690, CST, USA), Phospho-p38-MAPK (Thr180/Tyr182) (1:1000, #4511, CST, USA), p44/42 MAPK (1:1000, #4695, CST, USA), p-p44/42 MAPK (1:2000, #4370, CST, USA), SAPK/JNK (1:1000, #9252, CST, USA), p-SAPK/JNK (1:1000, #4668, CST, USA), and GAPDH (1:1000, #5174, CST, USA) at 4 °C overnight. The membranes were washed three times with 1x TBST (T1081, Solarbio, China) for 5 min each time and then incubated with secondary antibodies labeled with HRP at room temperature for 1 h. The bands were visualized using Omni-ECL™pico light chemiluminescence kits (Epizyme, China). GAPDH was used as the reference protein. All blots or gels derive from the same experiment and were processed in parallel. Original blots are provided in Supplementary Fig. 8.

### Bile tolerance

Bile salts were added to the BHI liquid medium (mass concentration; 0, 0.1, 0.3, and 0.5 g/100 mL) into test tubes. The tubes were autoclaved at 121 °C for 20 min, then cooled to room temperature. The activated *Bu* was inoculated with 5% inoculum into the above medium. The OD600 value of each group was measured after 24 h of anaerobic static culture at 37 °C. The survival rate was calculated as follows: (N1/N0) × 100%. N1 (log CFU/mL) was described as the total viable count of selected strains after treatment, while N0 (log CFU/mL) represents the total viable count of selected strains before treatment^70^.

### *B. uniformis* JCM5838 genome sequencing

Purified *B. uniformis* JCM5828 was cultured anaerobically at 37 °C for 24 h, then the cultured liquid (50 mL) was centrifuged at 12,000 × *g* and 4 °C for 10 min to collect the cell biomass. Genomic DNA of *B. uniformis* JCM5828 was extracted using a Wizard® Genomic DNA Purification Kit (Promega, USA). Purified genomic DNA was quantified using a TBS-380 fluorometer (Turner BioSystems Inc., Sunnyvale, CA, USA). High-quality DNA (OD260/280 = 1.8-2.0, ≥10 μg) was used for further research. Genomic DNA was sequenced using a combination of PacBio Sequel II and Illumina sequencing. The data generated from PacBio and Illumina platforms were used for bioinformatics analysis. All analyses were performed using the free online Majorbio Cloud Platform (http://cloud.majorbio.com) from Shanghai Majorbio Bio-pharm Technology Co., Ltd.

### Metagenomic sequencing and analysis

The colonic content samples were processed as follows. First, 16 frozen samples were thawed on ice, then the sample (50 mg) was put into a 2 mL centrifuge tube, and the true sample mass was recorded. Methanol internal standard solution (500 μL, 70%) was added to the sample, vortexed for 3 min, then allowed to stand in a −20 °C freezer for 30 min. The liquid was centrifuged at 12,000 × *g* and 4 °C for 10 min. The supernatant (250 μL) was aspirated and centrifuged at 12,000 × *g* and 4 °C for 5 min. The supernatant (200 μL) in the injection bottle was also aspirated and used for onboard analysis. Qualitative analysis was performed using the Metware database (MWDB) based on the retention time (RT) of the test substance, daughter ion pair information, and secondary spectral data of the test substance. Metabolite quantification was accomplished by multiple reaction monitoring analysis using triple quadrupole mass spectrometry. Mass spectrometry data were processed using Analyst 1.6.3 (Sciex, USA).

### Isolation of lamina propria lymphocytes

The intestines were cut open and rinsed with ice-cold PBS to isolate the colon lamina propria cells. Associated fats were removed and the colonic tissue was incubated in prewarmed 1× Hank’s Balanced Salt Solution at 37 °C for 40 min (without calcium and magnesium) containing dithiothreitol (1 mM), Ethylene Diamine Tetraacetic Acid (EDTA, 5 mM), and 1% fetal bovine serum (FBS) in a shaking incubator. The tissues were rinsed with warm Roswell Park Memorial Institute (RPMI) at 37 °C for 1 h containing 50 μg ml^−1^ Liberase D, 50 μg ml^−1^ DNase I, and 1% FBS to remove excess EDTA and digestion medium in a shaking incubator. Mononuclear cells were collected at the interface of a 40%/80% Percoll gradient (Solarbio, China). The cells were washed twice with PBS and counted. The test was continued if the cell viability was above 95%.

### In vitro T cell culture

Native CD4^+^ T cells were isolated from the colon lamina propria of C57BL/6J mice (aged 6–8 weeks) via fluorescence-activated cell sorting (FACS). Then, 96-well flat-bottom plates were precoated with 50 μl of anti-CD3e (145-2C11, Thermo Fisher Scientific, USA, 0.25 μg ml^−1^) antibodies at 37 °C for 2 h. The naive CD4^+^ T cells (1 × 10^6^) were seeded into T cell medium (RPMI supplemented with 10% fetal bovine serum, 25 mM glutamine, 55 μM 2-mercaptoethanol, 100 U mL^−1^ penicillin, 100 mg mL^−1^ streptomycin), after multiple washes with 1 × DPBS. Their T cell receptor downstream signaling pathways (TCR activation) were activated using soluble anti-CD28 (13-0281-82, Thermo Fisher Scientific, USA, 2 μg mL^−1^) antibodies. LPS (L8880, Solarbio, China, 10 μg/mL) or IL-6 (200-06, Peprotech, USA, 20 ng ml^−1^) and human TGF-β1 (100-21, Peprotech, USA, 0.3 ng mL^−1^) were added to the sample for TH17 cell differentiation. The cells were cultured at 37 °C for 2–4 d to increase the final yield.

### Cell culture and treatment

Bacterial supernatants, α-muricholic acid (2393-58-0, Macklin, China), isochenodeoxycholic (566-24-5, Leyan, China), hyodeoxycholic (83-49-8, Sigma-Aldrich, USA), and isolithocholic acid (1534-25-6, Yuanye, China) were added to the sample after TCR activation. The T cells were then cultured at 37 °C in a humidified 5% CO_2_ atmosphere. The bacterial supernatant was collected using centrifugation (12,000 × *g*, 10 min). The bile acids were dissolved in DMSO and then added to T cells through a 0.2-μm filter for 3 d. Finally, flow cytometry was used to analyze the percentage of Th17 cells.

### Flow cytometry

Lymphocytes isolated from the mouse colon or CD4^+^ T cells cultured in vitro were stained with LIVE/DEAD Fixable dye (L23105, Thermo Fisher Scientific, USA) to exclude dead cells. Lymphocytes were stained with surface marker antibodies anti-mouse CD3 (1:100, 551163, BD Biosciences, USA) and anti-mouse CD4 (1:100, 563151, BD Biosciences, USA) for 30 min at room temperature. Subsequently, the cells were washed with flow cytometry staining buffer (00-4222-26, Thermo Fisher Scientific, USA), fixed, permeabilized with the fixation and permeabilization solution (554722, BD Biosciences, USA), and intracellularly stained to determine the cytokine factors. The following antibodies were used at the indicated dilutions for staining: anti-mouse Foxp3 (1:100, 17-5773-80, BD Biosciences, USA), anti-mouse RORγt (1:100, 562607, BD Biosciences, USA). The samples were analyzed using a Flow Cytometer (BD Biosciences, USA). FlowJo software (Tree Star Inc., San Carlos, CA, USA) was used for subsequent analyses.

### Statistical analysis

The Wilcoxon rank-sum test, one-way ANOVA with Tukey’s test, and Kruskal–Wallis with Tukey–Kramer’s test were used to analyze the data. Microbial PCoA analysis was performed using Bray–Curtis, Unweighted-Unifrac, and Abund–Jaccard distance algorithms, and Adonis was used to test for differences between groups with 999 permutations. Microbial alpha diversity analysis, difference analysis at different taxonomic levels, and PICRUSt2 functional prediction analysis were performed using the Kruskal–Wallis *H*-test. All-against-all multi-group comparisons were used for LEfSe difference distinction analysis. A *P* value threshold of 0.05 (Wilcoxon rank-sum test) and an effect size threshold of 2 were used for all bacterial taxa. Metabolites with a VIP ≥1 were selected as differentially significant. Data analysis was carried out using SPSS (version 26.0; (SPSS Inc., Chicago, IL, USA), and one-way ANOVA was used to analyze differences among groups. Tukey’s multiple comparison test was used for post hoc analysis, and all results were expressed as mean ± SEM. Refer to the figure legend for specific analysis methods. A *P* value <0.05 was considered statistically significant, and the level of significance is denoted by asterisks in the figures (*P < 0.05, **P < 0.01, ***P < 0.001). The graphs were plotted using GraphPad Prism 9.0 software (La Jolla, CA, USA).

### Reporting summary

Further information on research design is available in the Nature Research Reporting Summary linked to this article.

### Supplementary information

Supplementary Information

Supplementary Table

Reporting Summary

## Data Availability

The samples of 16S rRNA gene sequencing is available from the NCBI under accession PRJNA 911642 and RNA-Seq sequencing is available from the NCBI under accession PRJNA912399. The whole genome sequencing is available from the NCBI under accession CP129143.
