# Supplementary material for: Bacteroides uniformis-induced perturbations in colonic microbiota and bile acid levels inhibit TH17 differentiation and ameliorate colitis developments
Source: NPJ Biofilms Microbiomes. 2023 Aug 14;9:56. doi: 10.1038/s41522-023-00420-5 (PMC10425470; doi:10.1038/s41522-023-00420-5)
Supplement: Supplementary file 1 — Supplementary Information [file 41522_2023_420_MOESM1_ESM.docx]

**Supplementary Information**

***Bacteroides uniformis*-induced perturbations in colonic microbiota and bile acid levels inhibit TH17 differentiation and ameliorate colitis development**

YiTing Yan^1†^, Yu Lei^1†^, Ying Qu^1^, Zheng Fan^1^, Ting Zhang^1^, Yangbin Xu^1^, Qian Du^2^, Daniel Brugger^3^, Yulin Chen^1^, Ke Zhang^1^*, Enping Zhang^1^*

^†^These authors contributed equally to this work.

*Correspondence authors.

Email addresses: zhangenping@nwafu.edu.cn (E.Z); kezhang@nwafu.edu.cn (K.Z)

**
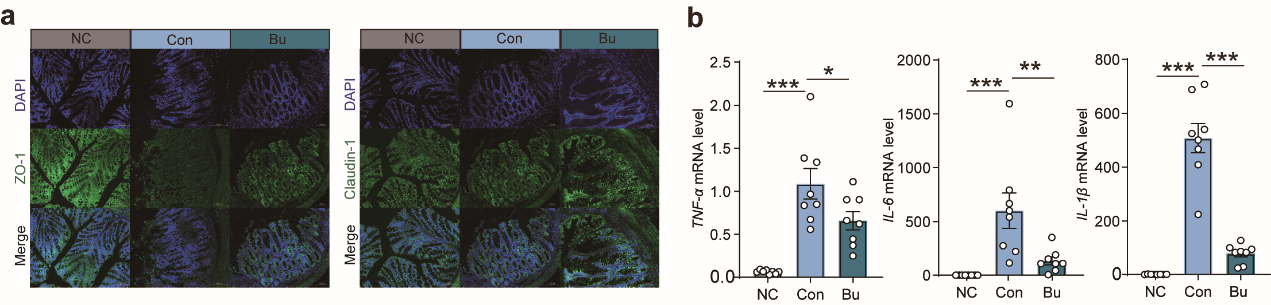
**

**Supplementary Fig. 1** ***B. uniformis* JCM5828 ameliorate DSS-induced colitis by strengthening the mechanical barrier and inhibiting the secretion of pro-inflammatory cytokines.** (a) Immunofluorescence staining of *ZO-1* and Claudin-1 proteins in colon tissues (Scale bar = 100 μm, *n* = 8). (b) qPCR analysis showing the mRNA expression of *IL-6*, *IL-1β*, *TNF-α* in colon tissues (*n* = 8). **p* < 0.05, ***p* < 0.01, ****p*< 0.001. Data were analyzed using one-way ANOVA with Tukey’s test and expressed as the means ± SEM.

**Supplementary Fig.
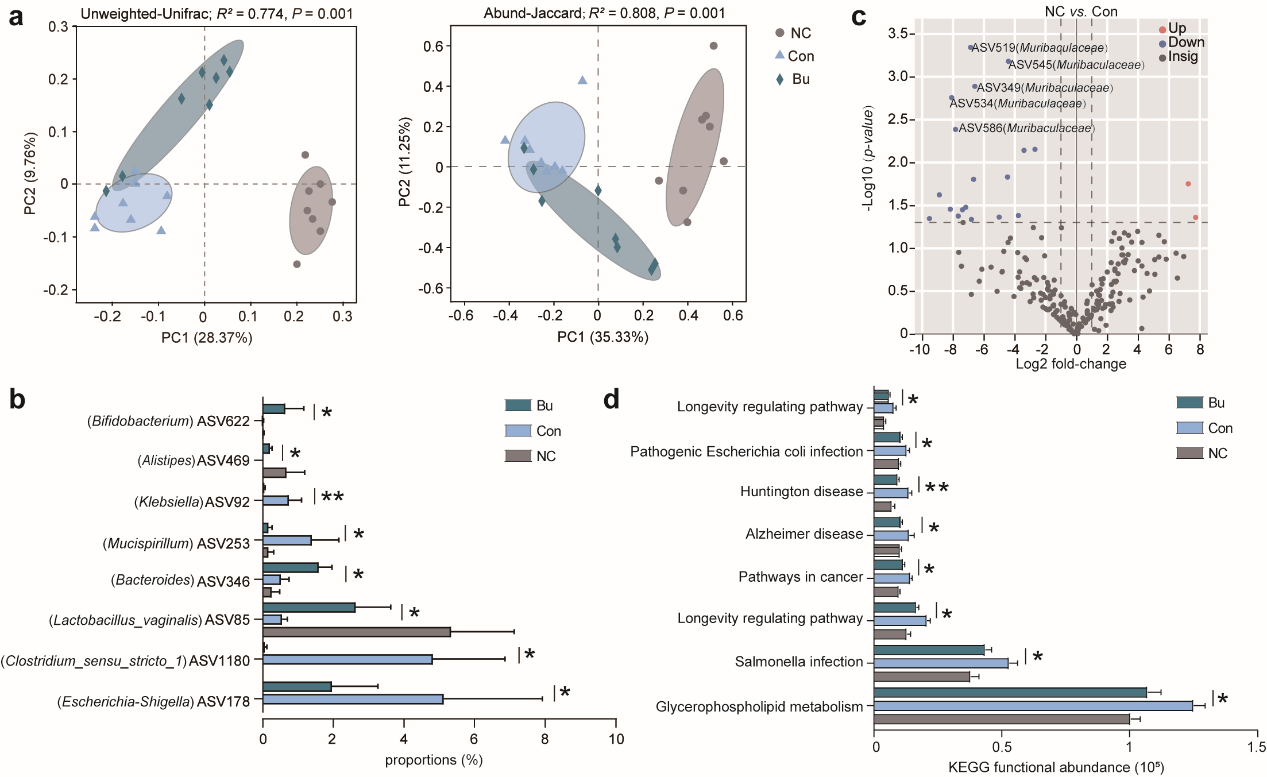
2 *B. uniformis* JCM5828 ameliorates DSS-induced colitis by reshaping gut microbiota.** (a) Principle coordinate analysis (PCoA) plot based on the ASV matrix in three groups. β-diversity was determined using Adonis with Unweighted-Unifrac and Abund-Jaccard test. (b) Bacterial ASV (relative abundance in the top 60) showing significant differences in their relative abundance between the Con and Bu group. Data were analyzed using Kruskal-Wallis with Wilcoxon’s test. (c) Differentially enriched bacteria between NC and Con groups. Red or blue dots represent bacteria with higher or lower abundance in the Con group samples. Grey dots represent bacteria with no significant difference between the two groups. *P values* were adjusted by Benjamini & Hochberg (BH) method to control FDR. FDR-adjusted *P* < 0.05 was shown. (d) The potential functional pathways of colonic content microbiota based on PICRUSt2. Data differences were assessed using Kruskal-Wallis with Tukey-Kramer’s test. **p*< 0.05, ***p* < 0.01. Data were expressed as the means ± SEM.

**Supplementary Fig.
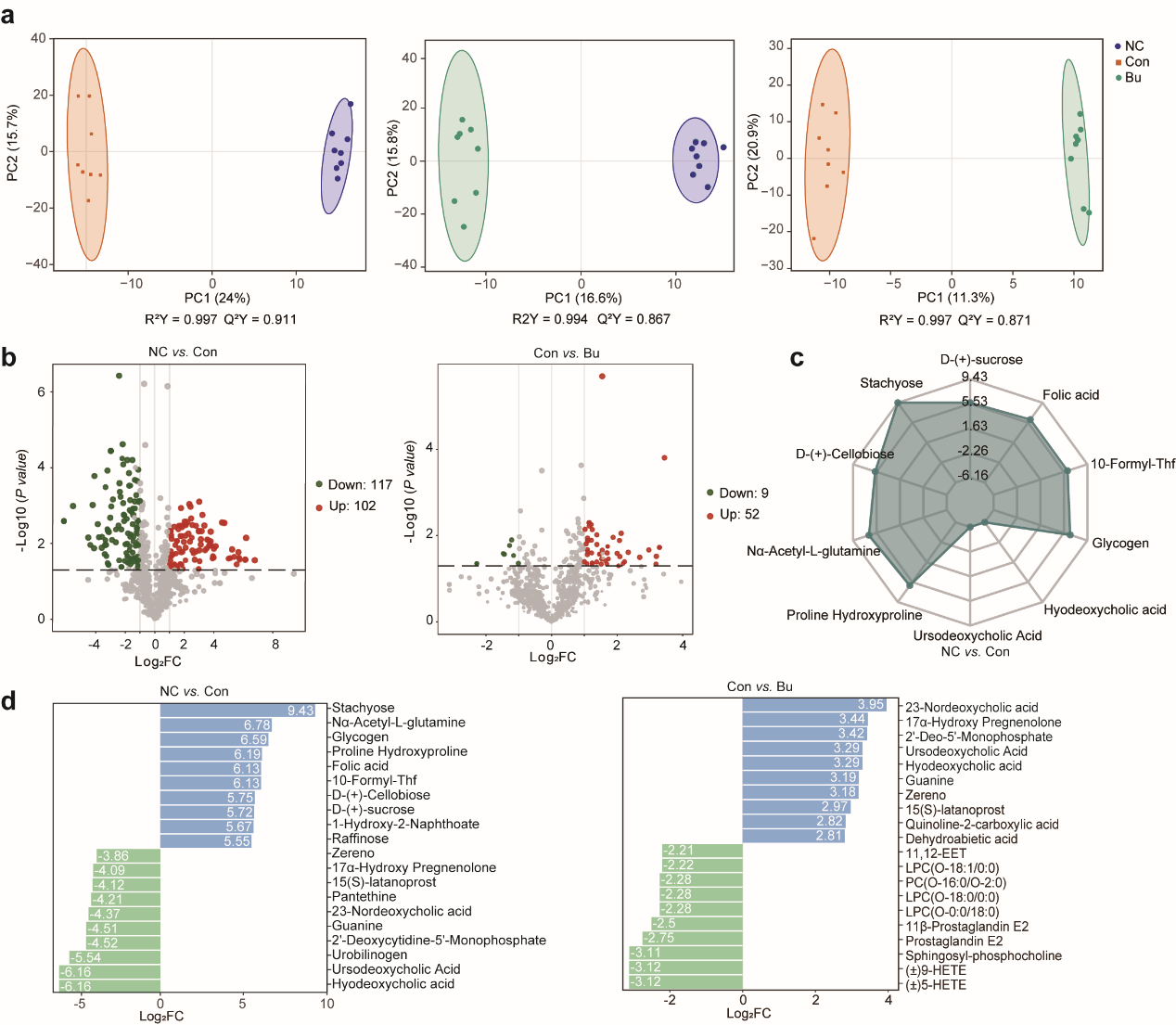
3** ***B. uniformis* JCM5828 restores DSS-mediated reduction in colonic bile acids abundance.** (a) OPLS-DA score plot of the colon contents. (b) Volcano plots for the RNA-Seq analyses of NC *vs*. Con and Con *vs*. Bu groups. The red dots represent differential metabolites upregulated in the Con or Bu groups, and the green dots represent differential metabolites downregulated in the Con or Bu groups. (c) Radar plot of differential metabolites in NC *vs.* Con groups. Grid lines represent log2 values of difference multiples. (d) Differential metabolites bar chart in NC *vs*. Con and Con *vs*. Bu groups. Blue bars represent differential metabolites upregulated in the Con or Bu groups, and green bars represent differential metabolites downregulated in the Con or Bu groups. The x-coordinate represents log2 value of the differential multiple.

**Supplementary Fig.
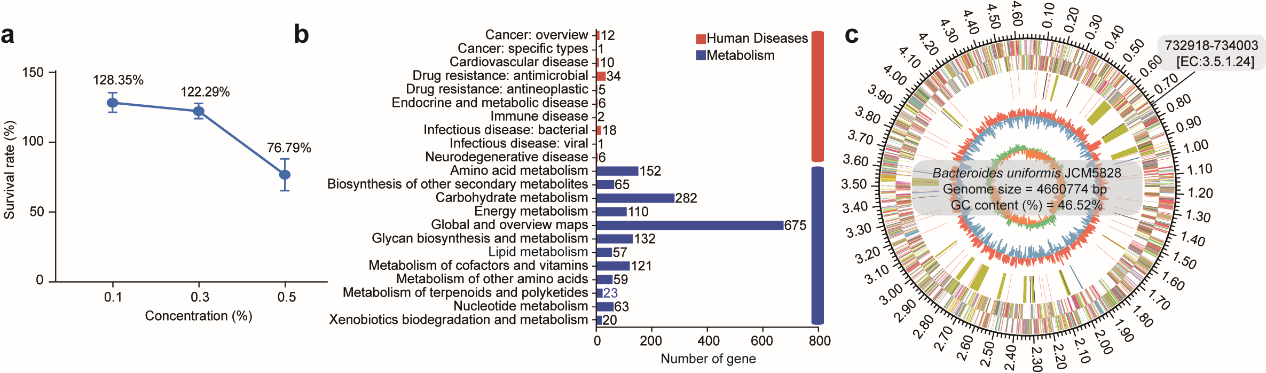
4 The key functional enzyme gene of *B. uniformis* is involved in the biosynthesis of bile acids.** (a) Survival rate of *B. uniformis* at three bile salt concentrations. Data were expressed as the means ± SEM. (b) KEGG pathway annotated classification statistics. The vertical and horizontal coordinates indicate level 2 hierarchical classification of the KEGG pathway and the number of genes under the annotated classification, respectively. The different bar colors represent level 1 classification of the KEGG pathway. (c) Genomic circos Map of *B. uniformis*. The outermost circle, second and third circle represent the genome size, CDS on positive and negative strands, respectively. Different colors indicate the functional classification of different COGs of CDS. The fourth circle represents rRNA and tRNA. The fifth circle represents GC content. The outward red part indicates that the GC content of the region is higher than the average GC content of the whole genome, and the higher peak indicates a larger difference. The inward blue part indicates that the GC content of the region is lower than the average GC content of the whole genome, and the higher peak indicates a larger difference. The innermost circle represents the GC-Skew value.

**
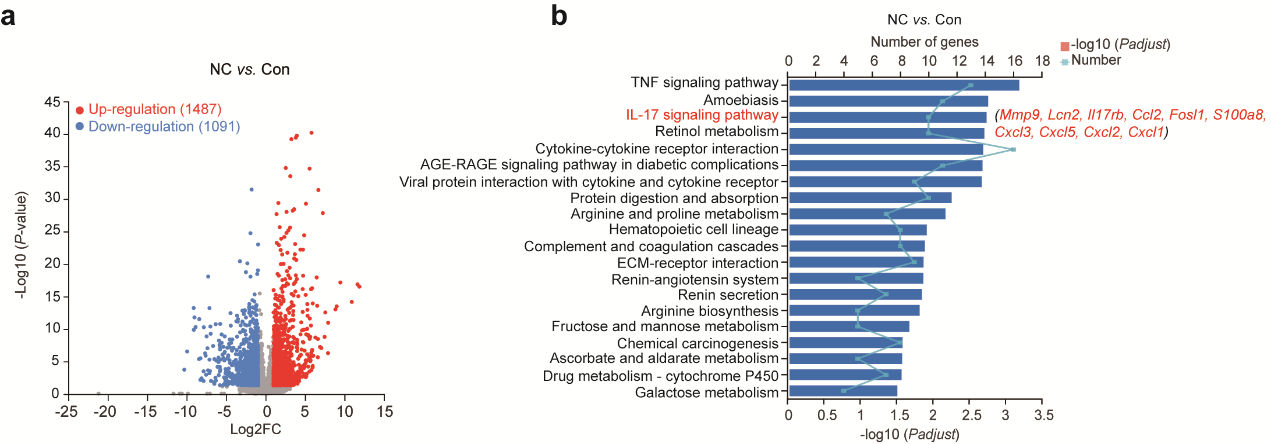
Supplementary Fig. 5 *B. uniformis* JCM5828 alleviates inflammation by inhibiting the IL-17 signaling pathway.** (a) Volcano plots for the RNA-Seq analyses of NC *vs.* Con group. The red and green dots represent up-regulated and downregulated DEGs in the Con group, respectively. (b) The KEGG enrichment analysis of NC *vs.* Con group. The red genes behind the bar represent DEGs in the Con group that are involved in the pathway and are upregulated.


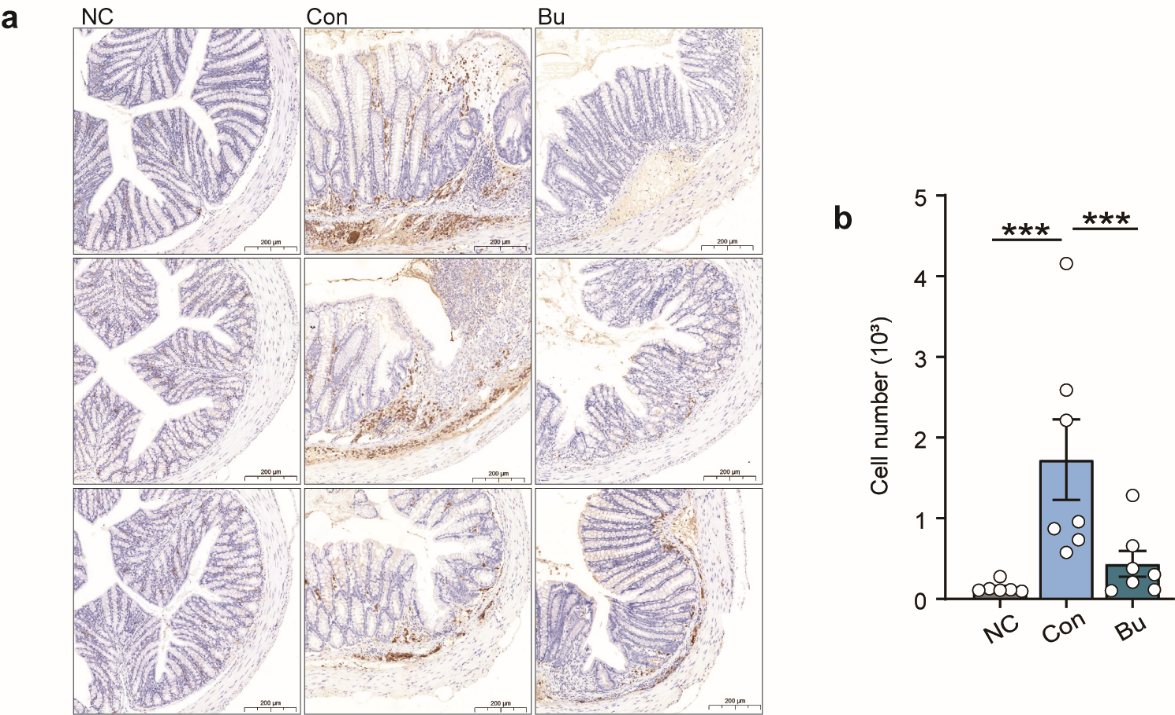


**Supplementary Fig. 6 Immunohistochemical staining of mouse colonic TH17 cells**. (a) Representative microscopic images of IL17A^+^ immunohistochemical staining of mouse colon tissue (Scale bar = 200 μm, *n* = 8). (b) The number of mouse colonic IL-17A^+^ cells (*n* = 8). ****p* < 0.001. Data were analyzed using one-way ANOVA with Tukey’s test and expressed as the means ± SEM.

**
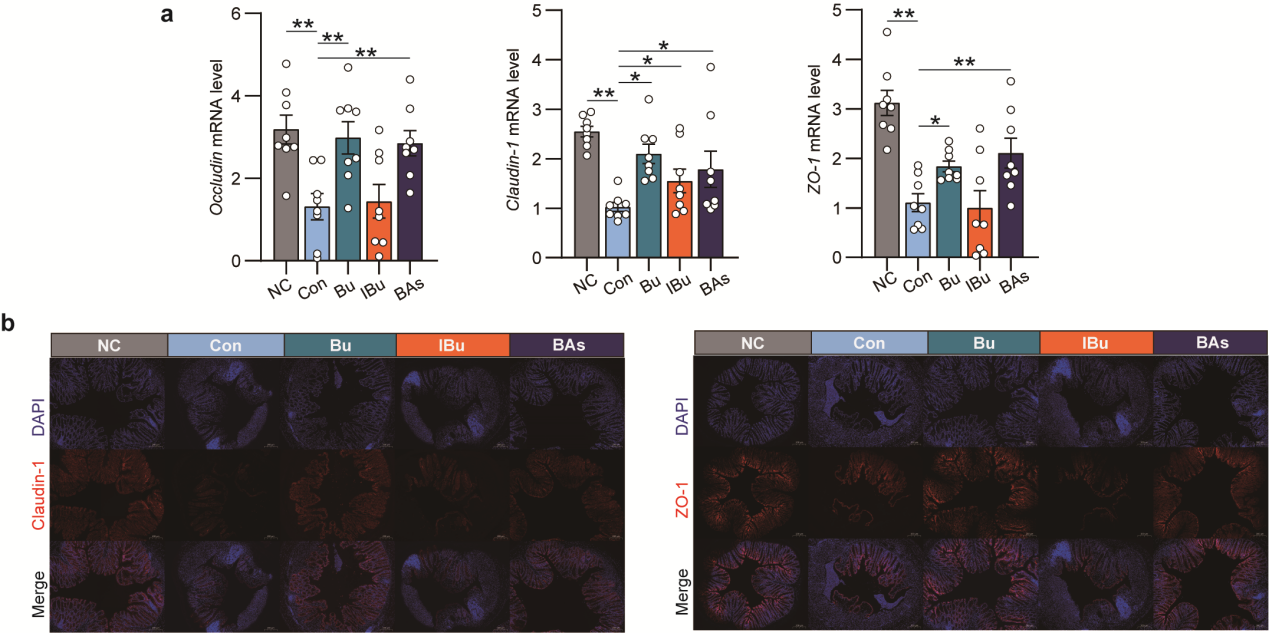
**

**Supplementary Fig. 7 Supplementation with *B. uniformis* JCM5828 or mixed bile acids restore the colonic mechanical barrier.** (a) qPCR analysis showing the mRNA expression of *Occludin*, *Claudin-1* and *ZO-1* in tissues (*n* = 8). Data were analyzed using one-way ANOVA with Tukey’s test. **p* < 0.05, ***p* < 0.01. (b) Immunofluorescence staining of Claudin-1 and *ZO-1* proteins in mouse colon (Scale bar = 200 μm, *n* = 8).


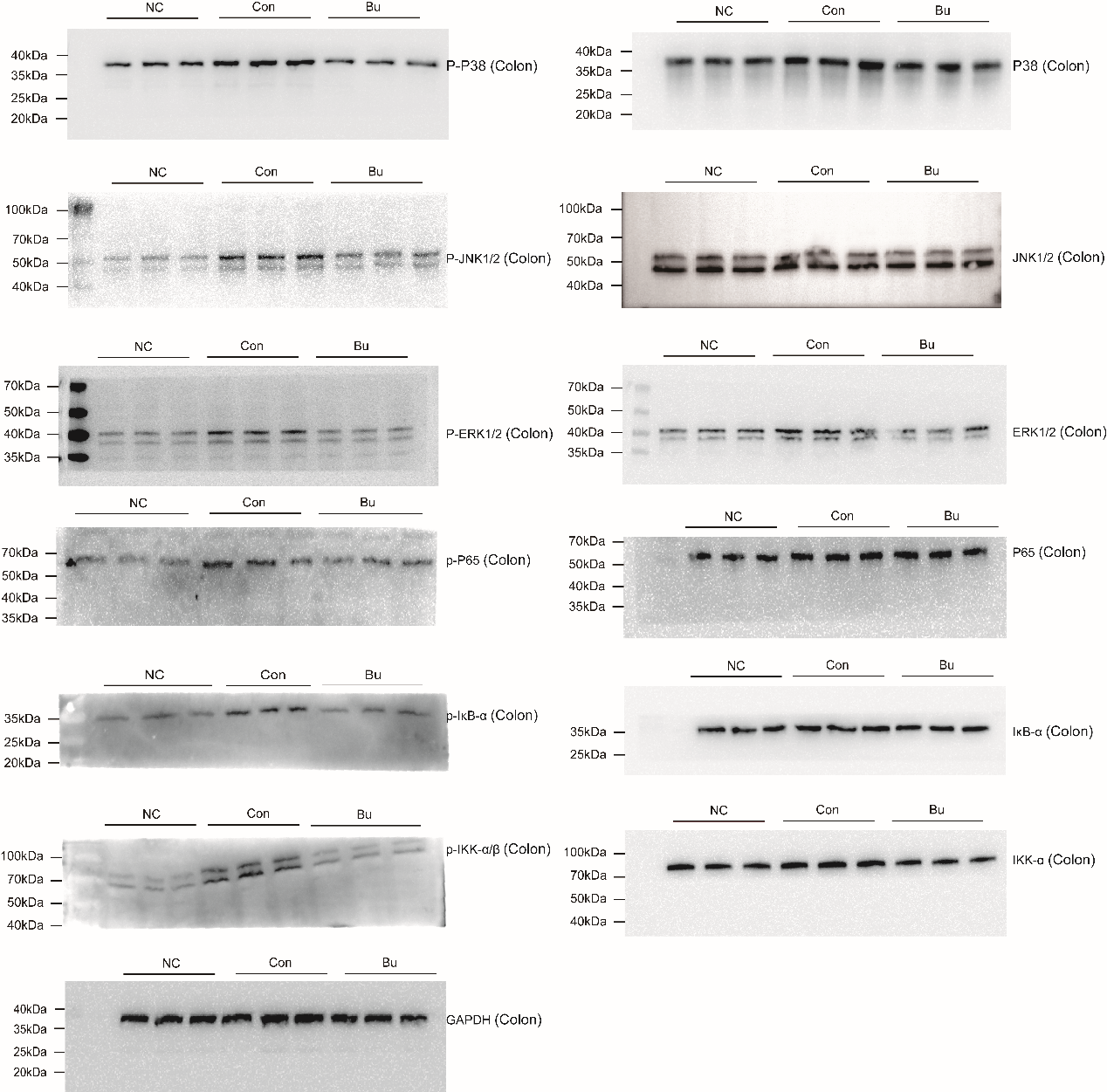
**Supplementary Fig. 8** **Original blots presented in the Fig. 3e.**

**Supplementary Table 1. The permutational PERMANOVA analysis. Related to Fig. 2b, Supplementary Fig. 2a.**

| **Distance Algorithm** | **Name** | **Df** | **SumsOfSqs** | **MeanSqs** | **F.Models** | **R2** | **Pr ( > F)** |
| --- | --- | --- | --- | --- | --- | --- | --- |
| bray-curtis | Base2 | 2 | 1.978789263 | 0.989394632 | 3.803597876 | 0.26591896 | 0.001 |
|  | Residuals | 21 | 5.462535195 | 0.260120724 | - | 0.73408104 | - |
|  | Total | 23 | 7.441324458 | - | - | 1 | - |
| unweighted-unifra | Base2 | 2 | 1.705894385 | 0.852947193 | 3.071275165 | 0.226307044 | 0.001 |
|  | Residuals | 21 | 5.832069769 | 0.277717608 | - | 0.773692956 | - |
|  | Total | 23 | 7.537964155 | - | - | 1 | - |
| abund-jaccard | Base2 | 2 | 1.736764051 | 0.868382025 | 2.502178481 | 0.192443019 | 0.001 |
|  | Residuals | 21 | 7.288058253 | 0.347050393 | - | 0.807556981 | - |
|  | Total | 23 | 9.024822304 | - | - | 1 | - |

**Supplementary Table 2. The relative abundance of top 8 bacterial ASVs. Related to Supplementary Fig. 2b.**

| **Species name** | **NC-mean (%)** | **NC-sd (%)** | **Con-mean (%)** | **Con-sd (%)** | **Bu-mean (%)** | **Bu-sd (%)** | ***p value* (NC vs Con)** | ***p value* (Con vs Bu)** |
| --- | --- | --- | --- | --- | --- | --- | --- | --- |
| ASV178 | 0 | 0 | 5.127 | 7.913 | 1.962 | 3.67 | 0.0004099 | 0.0486 |
| ASV1180 | 0 | 0 | 4.82 | 5.766 | 0.0608 | 0.1691 | 0.004569 | 0.02147 |
| ASV85 | 3.54 | 2.089 | 0.5499 | 0.4132 | 2.642 | 2.786 | 0.001948 | 0.04057 |
| ASV346 | 0.05096 | 0.0998 | 0.5212 | 0.6313 | 1.589 | 1.067 | 0.01587 | 0.02385 |
| ASV253 | 0.1609 | 0.4323 | 1.4 | 2.158 | 0.1627 | 0.3099 | 0.004729 | 0.01533 |
| ASV92 | 0 | 0 | 0.7439 | 0.9981 | 0.04649 | 0.06397 | 0.00146 | 0.009009 |
| ASV469 | 0.08583 | 0.1378 | 0.1153 | 0.2036 | 0.5946 | 0.5552 | 0.9553 | 0.01984 |
| ASV622 | 0.02414 | 0.03585 | 0.0152 | 0.04299 | 0.6428 | 1.492 | 0.4072 | 0.01767 |

**Supplementary Table 7. The dietary nutritional components of mice.**

| **Nutritive Index** | **Content** |
| --- | --- |
| Moisture（g/kg） | ≤100 |
| Crude protein（g/kg） | ≥200 |
| Crude fat（g/kg） | ≥40 |
| Crude fibre（g/kg） | ≤50 |
| Crude ash（g/kg） | ≤80 |
| Calcium（g/kg） | 10～18 |
| Total phosphorus（g/kg） | 6～12 |
| Amino acid | |
| Lysine（g/kg） | ≥13.2 |
| Methionine + Cystine（g/kg） | ≥7.8 |
| Arginine（g/kg） | ≥11.0 |
| Histidine（g/kg） | ≥5.5 |
| Tryptophan（g/kg） | ≥2.5 |
| Phenylalanine + Tyrosine（g/kg） | ≥13.0 |
| Threonine（g/kg） | ≥8.8 |
| Leucine（g/kg） | ≥17.6 |
| Valine（g/kg） | ≥11.7 |
| Isoleucine（g/kg） | ≥10.3 |
| Minerals | |
| Magnesium（g/kg） | ≥2.0 |
| Kalium（g/kg） | ≥5.0 |
| Natrium（g/kg） | ≥2.0 |
| Iron（mg/kg） | ≥120 |
| Manganese（mg/kg） | ≥75 |
| Cuprum（mg/kg） | ≥10.0 |
| Zinc（mg/kg） | ≥30.0 |
| Iodine（mg/kg） | ≥0.5 |
| Selenium（mg/kg） | 0.1～0.2 |
| Energy supply ratio | |
| Protein（%） | 22.8 |
| Fat（%） | 13.8 |
| Carbohydrate（%） | 63.4 |
| Total heat（Kcal/kg） | 3656 |

| **Gene Symbol** | **Forward（5’-3’）** | **Reverse（5’-3’）** | **Product Length** |
| --- | --- | --- | --- |
| *β-actin* | AGGGAAATCGTGCGTGACAT | GGAAAAGAGCCTCAGGGCAT | 172 |
| *TNF-α* | AGGCACTCCCCCAAAAGATG | TTTGCTACGACGTGGGCTAC | 250 |
| *IL-6* | AATAGTCCTTCCTACCCCAA | GCTTAGGCATAACGCACT | 167 |
| *IL-1β* | AACTGCACTACAGGCTCCGAGA | GCCACAGGTATTTTGTCGTTGCTT | 163 |
| *IL-10* | GCCGGGAAGACAATAACTGC | GCCTGGGGCATCACTTCTAC | 223 |
| *IL-17a* | TCCTCCACCGCAATGAGCAT | GGTTGGCACCATCACAAGGA | 153 |
| *ZO-1* | GCCGCTAAGAGCACAGCAA | GCCCTCCTTTTAACACATCAGA | 172 |
| Claudin-1 | GGCCTTGGCTGTACCTTACC | GGAGCACCTTATCCCCGTTT | 109 |
| Occludin | TTGAAAGTCCACCTCCTTACAGA | CCGGATAAAAAGAGTACGCTGG | 129 |

**Supplementary Table 8. The primer sequences for quantitative real-time PCR analysis.**
