# Supplementary material for: Bacteroides uniformis-induced perturbations in colonic microbiota and bile acid levels inhibit TH17 differentiation and ameliorate colitis developments
Source: NPJ Biofilms Microbiomes. 2023 Aug 14;9:56. doi: 10.1038/s41522-023-00420-5 (PMC10425470; doi:10.1038/s41522-023-00420-5)
Supplement: Supplementary file 3 — Reporting Summary [file 41522_2023_420_MOESM3_ESM.pdf]

nature portfolio

Corresponding author(s): Professor Enping Zhang, Dr. Ke Zhang

Last updated by author(s): Jun 29, 2023

Reporting Summary

Nature Portfolio wishes to improve the reproducibility of the work that we publish. This form provides structure for consistency and transparency in reporting. For further information on Nature Portfolio policies, see our Editorial Policies and the Editorial Policy Checklist.

Statistics

For all statistical analyses, confirm that the following items are present in the figure legend, table legend, main text, or Methods section.

n/a	Confirmed	
☐	☒	The exact sample size (*n*) for each experimental group/condition, given as a discrete number and unit of measurement
☐	☒	A statement on whether measurements were taken from distinct samples or whether the same sample was measured repeatedly
☐	☒	The statistical test(s) used AND whether they are one- or two-sided<br>*Only common tests should be described solely by name; describe more complex techniques in the Methods section.*
☐	☒	A description of all covariates tested
☐	☒	A description of any assumptions or corrections, such as tests of normality and adjustment for multiple comparisons
☐	☒	A full description of the statistical parameters including central tendency (e.g. means) or other basic estimates (e.g. regression coefficient) AND variation (e.g. standard deviation) or associated estimates of uncertainty (e.g. confidence intervals)
☐	☒	For null hypothesis testing, the test statistic (e.g. *F*, *t*, *r*) with confidence intervals, effect sizes, degrees of freedom and *P* value noted<br>*Give P values as exact values whenever suitable.*
☒	☐	For Bayesian analysis, information on the choice of priors and Markov chain Monte Carlo settings
☒	☐	For hierarchical and complex designs, identification of the appropriate level for tests and full reporting of outcomes
☒	☐	Estimates of effect sizes (e.g. Cohen's *d*, Pearson's *r*), indicating how they were calculated

Our web collection on statistics for biologists contains articles on many of the points above.

Software and code

Policy information about availability of computer code

Data collection	The cells were sorted using a Flow Cytometer (BD Biosciences, US);<br><br>SlideViewer 2.5.0 (DHISTECH Ltd., Hungary) was used for imaging.
Data analysis	The routine mice experiments data analysis was carried out using SPSS 26.0 (SPSS Inc., Chicago, IL, USA), The graphs were plotted using GraphPad Prism 9.0 software (La Jolla, CA, USA);<br><br>Flow Cytometer (BD Biosciences, US) and Flowjo software (Tree Star Inc., San Carlos, CA) were used for cell analyses;<br><br>Metabolite quantification is accomplished by multiple reaction monitoring (MRM) analysis using triple quadrupole mass spectrometry. The mass spectrometry data were processed using the software Analyst 1.6.3;<br><br>16S rRNA, RNA-Seq and whole genome sequencing were performed by Majorbio Biotechnology Ltd. The analysis results are described online on the Majorbio cloud platform (www.majorbio.com);<br><br>Image J software (National Institutes of Health, Bethesda, Maryland) was used to analyze the fluorescence signal intensity and the cell numbers.

For manuscripts utilizing custom algorithms or software that are central to the research but not yet described in published literature, software must be made available to editors and reviewers. We strongly encourage code deposition in a community repository (e.g. GitHub). See the Nature Portfolio guidelines for submitting code & software for further information.

Data

Policy information about availability of data

All manuscripts must include a data availability statement. This statement should provide the following information, where applicable:

- Accession codes, unique identifiers, or web links for publicly available datasets
- A description of any restrictions on data availability
- For clinical datasets or third party data, please ensure that the statement adheres to our policy

All raw and processed data will be made available upon request.
The samples of 16S rRNA gene sequencing was available from the NCBI under accession PRJNA 911642, RNA-Seq sequencing was available from the NCBI under accession PRJNA912399, and the whole genome sequencing was available from the NCBI under accession PRJNA954310.

Research involving human participants, their data, or biological material

Policy information about studies with human participants or human data. See also policy information about sex, gender (identity/presentation), and sexual orientation and race, ethnicity and racism.

Reporting on sex and gender	n/a
Reporting on race, ethnicity, or other socially relevant groupings	n/a
Population characteristics	n/a
Recruitment	n/a
Ethics oversight	n/a

Note that full information on the approval of the study protocol must also be provided in the manuscript.

Field-specific reporting

Please select the one below that is the best fit for your research. If you are not sure, read the appropriate sections before making your selection.

☒ Life sciences ☐ Behavioural & social sciences ☐ Ecological, evolutionary & environmental sciences

For a reference copy of the document with all sections, see nature.com/documents/nr-reporting-summary-flat.pdf

Life sciences study design

All studies must disclose on these points even when the disclosure is negative.

Sample size	For quantitative experiments with all phenotypic characterization data from mice, microbial data and metabolomic data, n=8 was chosen as the minimum number of replicates; for flow cytometry and Western blot analysis, n=3 was chosen as the minimum number of replicates. We considered this to be sufficient due to the internal control (specific staining of identified cell types using known cell markers) and the low variability observed between stained samples.
Data exclusions	At the beginning of the experiment, we set up 16 replicates for each group of mice, and because continuous treatment with 3% DSS for 24 days would result in a 50% mortality rate in one group of mice, eight mice per group were uniformly selected for subsequent experiments.
Replication	All replication attempts were successful. In a second mouse experiment, the therapeutic effect of the target strain and metabolites on DSS-induced colitis was verified, in agreement with the previous results, as shown in Figure 5. The transcriptome sequencing results (inflammatory pathway proteins) were verified by western blotting, in agreement with the transcriptome results. Differentiation of immune cells by target strains or metabolites was detected by flow cytometry, and the results were the same on both occasions.
Randomization	The allocation used for each group of mice was randomly selected. All fecal and tissue samples that passed quality control were analyzed equally with no sub-sampling and thus, there was no requirement for randomization.
Blinding	For the Disease Activity Index (DAI) and slice staining statistics, we chose two independent investigators blinded to assess these surrogate subjective outcomes.

Reporting for specific materials, systems and methods

We require information from authors about some types of materials, experimental systems and methods used in many studies. Here, indicate whether each material, system or method listed is relevant to your study. If you are not sure if a list item applies to your research, read the appropriate section before selecting a response.

Materials & experimental systems

n/a	Involved in the study	
☐	☒	Antibodies
☒	☐	Eukaryotic cell lines
☒	☐	Palaeontology and archaeology
☐	☒	Animals and other organisms
☒	☐	Clinical data
☒	☐	Dual use research of concern
☒	☐	Plants

Methods

n/a	Involved in the study	
☒	☐	ChIP-seq
☐	☒	Flow cytometry
☒	☐	MRI-based neuroimaging

Antibodies

Antibodies used	Anti-ZO-1 (ab221547, Abcam, UK)<br>Anti-Claudin-1 (ab211737, Abcam, UK)<br>Anti-DAPI (C0060, solarbio, China)<br>Anti-IKK-α (ab32041, Abcam, UK)<br>Anti-Phospho-IKKα/β (Ser176/180) (#2697, CST, US)<br>Anti-IKB-α (ab32518, Abcam, UK)<br>Anti-IKB-α (phospho S36) (ab133462, Abcam, UK)<br>Anti-NF-κB p65 (#8242, CST, US)<br>NF-κB p65(phospho Ser536) (ab76302, Abcam, UK)<br>Anti-p38 MAPK (#8690, CST, US)<br>Anti-Phospho-p38 MAPK (Thr180/Tyr182) (#4511, CST, US)<br>Anti-p44/42 MAPK (#4695, CST, US)<br>Anti-p-p44/42 MAPK (#4370, CST, US)<br>Anti-SAPK/JNK (#9252, CST, US)<br>Anti-p-SAPK/JNK (#4668, CST, US)<br>Anti-GAPDH (#5174, CST, US)<br>Anti-IL17A (eBio17B7, eBioscienceTM, US)<br>Anti-CD3e (145-2C11, Thermo, US,)<br>Anti-CD28 (13-0281-82, Thermo, US,)<br>Anti-CD3 (551163, BD Biosciences, US)<br>Anti-CD4 (563151, BD Biosciences, US)<br>Anti-Foxp3 (17-5773-80, eBiosci-ence, US)<br>Anti-RORγt (562607, BD Biosciences, US)
Validation	Anti-ZO-1 (ab221547, Abcam, UK) and Anti-Claudin-1 (ab211737, Abcam, UK) have been validated for use in immunofluorescence, as stated on the Abcam product page.<br><br>Anti-IKK-α (ab32041, Abcam, UK), Anti-Phospho-IKKα/β (Ser176/180) (#2697, CST, US), Anti-IKB-α (ab32518, Abcam, UK), Anti-IKB-α (phospho S36) (ab133462, Abcam, UK), Anti-NF-κB p65 (#8242, CST, US), NF-κB p65(phospho Ser536) (ab76302, Abcam, UK), Anti-p38 MAPK (#8690, CST, US), Anti-Phospho-p38 MAPK (Thr180/Tyr182) (#4511, CST, US), Anti-p44/42 MAPK (#4695, CST, US), Anti-p-p44/42 MAPK (#4370, CST, US), Anti-SAPK/JNK (#9252, CST, US), Anti-p-SAPK/JNK (#4668, CST, US) and Anti-GAPDH (#5174, CST, US)have been validated for use in western blotting, as stated on the Abcam or Cell Signaling Technology products page.<br>Anti-IL17A (eBio17B7, eBioscienceTM, US) has been validated for use in western blotting, as stated on the Thermo Fisher product page.<br><br>Anti-CD3e (145-2C11, Thermo, US) and Anti-CD28 (13-0281-82, Thermo, US) have been validated for use in T-cell activation, as stated on the Thermo Fisher product page.<br><br>Anti-CD3 (551163, BD Biosciences, US), Anti-CD4 (563151, BD Biosciences, US), Anti-Foxp3 (17-5773-80, eBiosci-ence, US) and Anti-RORγt (562607, BD Biosciences, US) have been validated for use in flow cytometry, as stated on the Thermo Fisher product page.

Animals and other research organisms

Policy information about studies involving animals; ARRIVE guidelines recommended for reporting animal research, and Sex and Gender in Research

Laboratory animals	Wild-type C57BL/6J female mice aged 8 weeks old were used for the experiments.
Wild animals	No wild animals were used in this study.
Reporting on sex	The findings do not apply to only one sex, and only female C57BL/6J mice were used in our experiments; the reason for the lack of gender-based analysis was to avoid the interference of other factors arising from sex differences and to refer to the fact that most of

the literature on microbially treated mice used female mice as subjects.

Field-collected samples: No field-collected samples were used in this study.

Ethics oversight: The experiment was approved by the Institutional Animal Care and Use Committee of the Northwest A&F University (permit number: 2021-06-008 and 2023-02-010).

Note that full information on the approval of the study protocol must also be provided in the manuscript.

Flow Cytometry

Plots

Confirm that:

☒ The axis labels state the marker and fluorochrome used (e.g. CD4-FITC).

☒ The axis scales are clearly visible. Include numbers along axes only for bottom left plot of group (a 'group' is an analysis of identical markers).

☒ All plots are contour plots with outliers or pseudocolor plots.

☒ A numerical value for number of cells or percentage (with statistics) is provided.

Methodology

Sample preparation: The intestines were cut open and rinsed using ice-cold PBS to isolate the colon lamina propria cells. Associated fats were carefully removed and incubated in prewarmed 1× HBSS (at 37 °C for 40 min) (without calcium and magnesium) containing 1 mM dithiothreitol, 5 mM EDTA and 1% fetal bovine serum (FBS) in a shaking incubator. The tissues were then rinsed with warm RPMI (at 37 °C for 1 h) containing 50 µg ml−1 Liberase D, 50 µg ml−1 DNase I and 1% FBS) to remove excess EDTA and digestion medium in a shaking incubator. Mononuclear cells were collected at the interface of a 40%/80% Percoll gradient (Solarbio, China). The cells were washed twice using PBS and counted. The test was continued if the cell viability was above 95%.
Native CD4+ T cells were isolated from the colon lamina propria of C57BL/6J mice (aged 6 to 8 weeks) via FACS sorting. Then, 96-well flat-bottom plates were precoated with 50 µl of anti-CD3e (145-2C11, Thermo, US, 0.25 µg ml−1) antibodies at 37 °C for 2 h. The naive CD4+ T cells (1×106) were seeded into T cell medium (RPMI supplemented with 10% fetal bovine serum, 25 mM glutamine, 55 µM 2-mercaptoethanol, 100 U ml−1 penicillin, 100 mg ml−1 streptomycin) after multiple washes with 1× DPBS. Their T cell receptor downstream signaling pathways (TCR activation) were activated with soluble anti-CD28 (13-0281-82, Thermo, US, 2 µg ml−1) antibodies. LPS (10 µg/mL) or IL-6 (200-06, Peprotech, US, 20 ng ml−1) and human TGF-β1 (100-21, Peprotech, US, 0.3 ng ml−1) were added to the sample for TH17 cell differentiation. The cells were cultured at 37 °C for 2-4 days to increase the final yield.
Bacterial supernatants, α-Muricholic acid (2393-58-0, Macklin, China), Isochenodeoxycholic acid (566-24-5, Leyan, China), Hyodeoxycholic acid (83-49-8, Sigma-Aldrich, US) and isolithocholic acid (1534-25-6, Yuanye, China) were added to the sample after TCR activation. The T cells were then cultured at 37 °C in humidified 5% CO2 atmosphere. The bacterial supernatant was collected via centrifugation (12,000 * g, 10 min). The bile acids were dissolved in DMSO, then added to T cells through a 0.2 µm filter for co-culture for three days. Finally, flow cytometry was used to analyze the percentage of Th17 cells.

Instrument: The samples were collected using a Flow Cytometer (BD Biosciences, US).

Software: Flowjo software (Tree Star Inc., San Carlos, CA) was used for subsequent analyses.

Cell population abundance: Experiment 1: lymphocytes (90.6%), CD4+ T cells (CD4+,98.5%), TH17 cells (RORγt+Foxp3-).
Experiment 2: lymphocytes (92.5%), CD4+ T cells (CD4+,93.2%), TH17 cells (RORγt+Foxp3-).
Experiment 3: lymphocytes (66.7%), TH17 cells (CD4+CD3+RORγt+).

Gating strategy: Lymphocytes isolated from mouse colon were stained with LIVE/DEAD Fixable dye (L23105, Thermo, US) to exclude dead cells. CD4+ T cells (CD4+CD3+) were labeled by staining lymphocytes with a surface marker antibodies for 30 minutes at room temperature. Subsequently, intracellular staining was performed to identify TH17 cells (RORγt+Foxp3-).

☒ Tick this box to confirm that a figure exemplifying the gating strategy is provided in the Supplementary Information.
